# A spatial version of the Stroop task for examining proactive and reactive control independently from non-conflict processes

**DOI:** 10.3758/s13414-024-02892-9

**Published:** 2024-04-30

**Authors:** Giacomo Spinelli, Stephen J. Lupker

**Affiliations:** 1 Dipartimento di Psicologia, Università degli Studi di Milano-Bicocca, Piazza dell’Ateneo Nuovo 1, 20126 Milano, MI Italy; 2 Department of Psychology, University of Western Ontario, London, Ontario N6A 5C2 Canada

**Keywords:** Conflict-induced control, Proactive control, Reactive control, Stroop, Proportion-congruent effect

## Abstract

Conflict-induced control refers to humans’ ability to regulate attention in the processing of target information (e.g., the color of a word in the color-word Stroop task) based on experience with conflict created by distracting information (e.g., an incongruent color word), and to do so either in a proactive (preparatory) or a reactive (stimulus-driven) fashion. Interest in conflict-induced control has grown recently, as has the awareness that effects attributed to those processes might be affected by conflict-unrelated processes (e.g., the learning of stimulus-response associations). This awareness has resulted in the recommendation to move away from traditional interference paradigms with small stimulus/response sets and towards paradigms with larger sets (at least four targets, distractors, and responses), paradigms that allow better control of non-conflict processes. Using larger sets, however, is not always feasible. Doing so in the Stroop task, for example, would require either multiple arbitrary responses that are difficult for participants to learn (e.g., manual responses to colors) or non-arbitrary responses that can be difficult for researchers to collect (e.g., vocal responses in online experiments). Here, we present a spatial version of the Stroop task that solves many of those problems. In this task, participants respond to one of six directions indicated by an arrow, each requiring a specific, non-arbitrary manual response, while ignoring the location where the arrow is displayed. We illustrate the usefulness of this task by showing the results of two experiments in which evidence for proactive and reactive control was obtained while controlling for the impact of non-conflict processes.

## Introduction

It has long been known that some form of control is required in human goal-oriented behavior in order to prevent distractions from disrupting that behavior (e.g., Miller & Cohen, 2001). In recent years, there has been an increasing interest in the *dynamic* nature of the relevant control processes (e.g., Chiu & Egner, 2019). According to these ideas, humans are able to regulate attention when processing a task-relevant stimulus component, or target (e.g., the color of a word in the color-word Stroop (1935) task), based on, most typically, experience with conflicting information created by a task-irrelevant stimulus component, or distractor (e.g., an incongruent color word).^1^ According to the Dual-Mechanisms of Control framework (Braver, 2012), this conflict-induced attention regulation can occur in two ways. First, it can occur *proactively* when conflict is anticipated and selective attention in processing target information (e.g., the color in a Stroop stimulus) is increased in a preparatory fashion. Second, it can occur *reactively*, with selective attention being regulated “on the fly” in response to irrelevant but potentially distracting information (e.g., an incongruent word).

Popular paradigms used to examine proactive and reactive control are Proportion-Congruent (PC) paradigms (Bugg & Crump, 2012). These paradigms involve contrasting Mostly-Congruent (MC) situations, in which most of the experimental trials are congruent (e.g., the word RED in the color red), with Mostly-Incongruent (MI) situations, in which most of the experimental trials are incongruent (e.g., the word RED in the color blue). In “list-wide” PC paradigms, the two situations being contrasted are two lists of trials, i.e., an MC list mostly composed of congruent stimuli and an MI list mostly composed of incongruent stimuli. The typical result is a larger congruency effect (i.e., the performance difference between incongruent and congruent trials) in the MC list than in the MI list (e.g., Logan & Zbrodoff, 1979).

This list-wide PC effect has often been interpreted (e.g., Botvinick et al., 2001) as being the result of a process of control adjustment occurring *in advance of stimulus presentation –* an item-nonspecific, proactive form of control. More specifically, proactive control would be engaged in the MI list, a type of list in which the high frequency of incongruent distractors, distractors that are assumed to produce conflict, leads individuals to anticipate conflict and prepare for it by increasing selective attention before the stimulus appears. The result is a reduced congruency effect in that situation. In the MC list, on the other hand, the low frequency of conflict creates little anticipation and, therefore, little advanced preparation for conflict. Hence, conflict, when it arises, must be dealt with at the time that it occurs – a reactive form of control. The result is an increased congruency effect in that situation. Therefore, although in many accounts, proactive and reactive control are both involved in the list-wide PC effect (see, e.g., De Pisapia & Braver, 2006), the overall effect is typically interpreted as mainly reflecting the action of an item-nonspecific, proactive control process adjusting attention based on the frequency of conflict in the two lists (e.g., Gonthier et al., 2016).

In the other types of PC paradigms, referred to as “item-specific” and “context-specific” PC paradigms, the two situations being contrasted are two sets of stimuli intermixed within the same list of trials. In the item-specific PC paradigms, the two sets are defined by the identities of targets and distractors, with one set composed of, for example, the colors red and blue mainly presented with their congruent words (the MC set) and another set composed of, for example, the colors green and yellow mainly presented with incongruent words (the MI set). The typical result, similar to what is observed in the list-wide PC paradigm, is a larger congruency effect for MC items than for MI items (e.g., Jacoby et al., 2003).

This item-specific PC effect has often been interpreted (e.g., Bugg et al., 2011) as being the result of a reactive process whereby, on one hand, the high frequency of conflict produced by distractors in MI items causes selective attention to be increased to better handle that conflict *when a particular item (i.e., a distractor and/or target) of that type is presented,* resulting in a reduced congruency effect for that type of item. The low frequency of conflict produced by distractors in MC items, on the other hand, does not cause a selective-attention increase when a particular item of that type is presented, resulting in an increased congruency effect for that type of item. Therefore, the item-specific PC effect would reflect the action of an item-specific, reactive process adjusting attention based on the frequency of conflict associated with the two types of items.

In the context-specific PC paradigms, the two sets being contrasted are defined by a task-irrelevant and non-interfering feature (e.g., the positioning of colored words above or below fixation, with the words’ position having virtually no impact on color-naming performance). Although this paradigm also tends to produce a PC effect (Crump et al., 2006), there is currently considerable controversy as to whether it really involves conflict-induced control (Bugg et al., 2020; Hutcheon, 2022; Hutcheon & Spieler, 2017; Schmidt & Lemercier, 2019; Weidler et al., 2022). For this reason, in the following, we mainly focus on list-wide and item-specific PC paradigms.

### Non-conflict processes can produce Proportion-Congruent (PC) effects

Although interest in list-wide and item-specific PC paradigms as tools for examining both proactive and reactive control has grown in recent years, so has the awareness that the effects produced using those paradigms might be affected by other processes (an awareness that, as discussed in the next section, has led to the development of new, less problematic paradigms). First, in list-wide PC paradigms, the list-wide PC manipulation can be confounded with an item-specific PC manipulation, such that all items in the MC list are MC items (i.e., all words in that list appear most often in their congruent color) and all items in the MI list are MI items (i.e., all words in that list appear most often in incongruent colors). Therefore, the process producing the list-wide PC effect in that type of situation might not be the item-nonspecific, proactive control process that is often assumed, but the same item-specific, reactive control process that is presumed to produce the item-specific PC effect in item-specific PC manipulations (Blais & Bunge, 2010; Blais et al, 2007).

Second, and most importantly for the present discussion, both list-wide and item-specific PC paradigms can contain confounds involving processes unrelated to conflict (Algom et al., 2022; Algom & Chajut, 2019; Schmidt, 2013a, 2019; Schmidt & Besner, 2008). One of those processes is the process of learning associations, or contingencies, between a stimulus and a motor response (Schmidt et al., 2007). That is, the words used in PC paradigms most frequently require the congruent response in MC situations and often require a particular *in*congruent response in MI situations. Because producing the typical, or high-contingency, response for a stimulus (e.g., the congruent response “red” for the MC word RED in an item-specific PC paradigm) is typically faster than producing an atypical, or low-contingency, response for that stimulus (e.g., the incongruent response “blue” for the MC word RED), the congruency effect in PC paradigms would be inflated by this contingency-learning process in MC situations, situations in which congruent responses are typically high-contingency responses. In contrast, if anything, the congruency effect would often be *de*flated in MI situations, situations in which *in*congruent responses are often high-contingency responses (Schmidt & Besner, 2008). Contingency learning would thus be capable of producing PC effects all by itself.

PC effects might also be affected by what might be called repetition-priming processes (Cochrane & Pratt, 2022a; Hazeltine & Mordkoff, 2014; Tzelgov et al., 1992; see also Schmidt et al., 2020). Responding to a stimulus is typically easier when the stimulus is repeatedly presented, either because, in a randomized list, both features of the stimulus (e.g., the color yellow and the word GREEN for GREEN in yellow) will tend to repeat more often from trial to trial (e.g., GREEN in yellow followed by another GREEN in yellow), allowing rapid retrieval of the required response (Hommel et al., 2004), or because of practice effects due to the accumulation of instances of the stimulus in memory (Logan, 1988). Because in PC paradigms, typically, each congruent stimulus is individually more frequent than any incongruent stimulus in MC situations (e.g., in an MC list, RED in red may be presented 36 times whereas RED in blue may only be presented 12 times), and vice versa in MI situations, repetition-priming effects would inflate the congruency effect in the former situations and deflate it in the latter. Therefore, like contingency learning processes, repetition-priming processes would be capable of producing PC effects all by themselves.

### Controlling for non-conflict processes in PC paradigms

In recent years, the rising awareness that in typical PC paradigms conflict-induced control processes are confounded with non-conflict processes (or other conflict-induced processes) has pushed researchers to develop alternative PC paradigms in which the targeted conflict-induced process can be observed while other processes, particularly non-conflict ones, are controlled (Blais & Bunge, 2010; Bugg, 2014; Bugg & Hutchison, 2013; Bugg et al., 2008; 2011; Hutchison, 2011; Schmidt, 2013c; Spinelli & Lupker, 2020, 2021; Spinelli et al., 2019). Those paradigms are the ones that most theorists in the area now recommend in order for conflict-induced control to be appropriately measured (Braem et al., 2019). In the list-wide PC paradigm, for example, the solution that has typically been adopted in order to control for non-conflict processes is to divide the stimuli used into two sets: an inducer set and a diagnostic set (also known as context and transfer sets, respectively). For inducer items, congruency proportion is manipulated directly as is typically done for all stimuli in traditional list-wide PC paradigms. This manipulation involves presenting, for example, two colors (e.g., red and blue) with their corresponding (congruent) words more often than with their noncorresponding (incongruent) words in the MC list (i.e., the MC inducer items), and the same two colors with their noncorresponding (incongruent) words more often than with their corresponding (congruent) words in the MI list (i.e., the MI inducer items). Although inducer items in this situation often produce a sizeable list-wide PC effect, this effect might result from either conflict-induced control and/or non-conflict processes (as is the case for any item in traditional list-wide PC paradigms in which no distinction is made between inducer and diagnostic items).

The same is not true for diagnostic items, however. For diagnostic items, congruency proportion is not manipulated as those items have a fixed 50:50 congruent/incongruent ratio. The diagnostic items, however, are intermixed with MC inducer items in the MC list (creating an overall MC list) and with MI inducer items in the MI list (creating an overall MI list). Because diagnostic items in the two lists are identical, non-conflict processes should have a similar direct impact, if they have any such impact at all, on those items in the two lists. Therefore, when a list-wide PC effect is observed for diagnostic items, that effect may not be attributed to non-conflict processes, at least, not to the direct effects of contingency-learning or repetition-priming processes. (There may, however, still be indirect effects of contingency learning (Bugg, 2014) or of other non-conflict processes involving temporal learning (Schmidt, 2013b) or of target-distractor correlation (Algom & Chajut, 2019), which may play some role on the emergence of the list-wide PC effect. Processes of this sort are discussed in the *General discussion*.) Instead, the effect can be attributed to a conflict-induced adjustment in proactive control increasing preparation for conflict in the MI list. Because a list-wide PC effect is indeed the effect that is typically reported in Stroop tasks for diagnostic items (although not always – for a discussion, see Spinelli & Lupker, 2023a), this list-wide PC paradigm appears to be an effective solution for examining conflict-induced control independently from non-conflict processes.

The designs adopted to control for non-conflict processes in the item-specific PC paradigm (e.g., Bugg et al., 2011; Bugg & Hutchison, 2013) have been more varied and, according to Schmidt (2019), most of them have not been completely successful at controlling for the impact of those processes. A design that appears to be among the least problematic is one that we recently developed (Spinelli & Lupker, 2020) based on a design previously introduced by Schmidt (2013c). This design does not involve a distinction between inducer and diagnostic items (or training and transfer items, as they are sometimes called in the context of item-specific PC manipulations: Bugg et al., 2011; Bugg & Hutchison, 2013), but, nonetheless, makes it possible to examine reactive control independently from non-conflict processes by contrasting MC incongruent stimuli and MI incongruent stimuli matched on contingency learning and individual stimulus frequency. A detailed explanation of the design for this contrast is described in the Introduction section of the present Experiment 2. The important point for now is that the fact that Spinelli and Lupker (2020) observed longer latencies for MC incongruent stimuli than for MI incongruent stimuli in that particular contrast can most likely be attributed to a reactive control process increasing selective attention to target information for the latter stimuli.

### The present research

Although different designs have been adopted to control for non-conflict processes in list-wide and item-specific PC paradigms, what those designs seem to have in common is the fact that they moved away from traditional interference paradigms with small stimulus/response sets (typically involving two targets, distractors, and responses) and towards paradigms with larger sets. For example, inducer/diagnostic designs require at least four targets, distractors, and responses (i.e., a set size of four) in the list-wide PC paradigm (of which at least two are assigned to the inducer items and two to the diagnostic items, e.g., Bugg et al., 2008) and six targets, distractors, and responses (i.e., a set size of six) in the item-specific PC paradigm, with some researchers recommending even higher minimums (e.g., Bugg & Gonthier, 2020). Spinelli and Lupker’s (2020) contingency-matching design for the item-specific PC paradigm, in particular, requires a set size of six because, in order to dissociate item-specific conflict frequency (i.e., the frequency of conflict associated with particular targets and/or distractors in the experiment) and contingency learning, the subsets of stimuli used for MC and MI items must not be overlapping and each of the two subsets needs a size of at least three (for a discussion of the reason for this requirement, see Spinelli & Lupker, 2020; for other item-specific PC paradigms that require a set size of six, see Bugg & Hutchison, 2013).

Using large sets of stimuli is not always feasible, however. Color-word and picture-word Stroop tasks do allow researchers to use larger stimulus sets because there are large numbers of nameable colors, pictureable objects, and interfering words, to choose from. Not surprisingly, those tasks are the main tasks in which the designs that allow control over non-conflict processes in PC paradigms have been implemented (Braem et al., 2019). Note that in those tasks, vocal responses are typically required, responses that, being non-arbitrary for colors and pictures, participants typically produce with ease. However, vocal responses can be difficult for researchers to collect, especially in neuroimaging research (in which any head motion must typically be avoided and, accordingly, vocal responses to Stroop stimuli have rarely been used)^2^ and in online experiments (in which remote use of voice keys is typically unsupported by relevant experimental software), with these types of experiments, particularly online experiments, becoming increasingly common in recent years (Arechar & Rand, 2021). In order to circumvent the problem that vocal responses pose, more recently several researchers have gone to using manual responses to colors and pictures in PC paradigms (e.g., Bejjani et al., 2020; Bejjani & Egner, 2021; Blais & Bunge, 2010; Chiu et al., 2017; Crump et al., 2017; Hutcheon, 2022).

Manual responses in color-word and picture-word Stroop tasks, however, have clear drawbacks. First and foremost, because manual responses are typically arbitrary for colors and pictures (but see below for tasks involving typing), they change the nature of the task from that of a proper Stroop task involving overlapping representations of relevant stimulus components (e.g., colors), irrelevant stimulus components (e.g., words), and responses (e.g., color name utterances), to that of a Stroop-*like* task involving overlapping representations of relevant and irrelevant stimulus components only (Kornblum, 1992; see also footnote 1 and, for a recent discussion of this problem, Viviani et al., 2023). The impact of such a change is reflected in the many reports of different patterns of results for vocal- versus manual-response Stroop tasks (e.g., Augustinova et al., 2019; Redding & Gerjets, 1977; Sharma & McKenna, 1998).

In addition to this change, the mere learning of arbitrary stimulus-response associations likely poses considerable difficulty for participants, a difficulty that may not be inconsequential. For example, learning and maintaining multiple arbitrary stimulus-response mappings throughout an experiment may create, in many cases, high working-memory demands that may prevent participants from applying proactive control, a resource-demanding control mode (Braver, 2012; see, e.g., Jiménez et al., 2021). In fact, for some populations with reduced cognitive abilities such as young children, multiple arbitrary stimulus-response mappings may create an excessive burden (see, e.g., Gonthier et al., 2021). Further, distractors (e.g., the word RED) likely do not produce a strong response conflict, that is, a tendency to produce their associated arbitrary responses (e.g., the key designated for the response “red”; MacLeod, 1991), whereas response conflict may be the most relevant conflict component for conflict-induced control (Spinelli & Lupker, 2023a).

Overall, at present, with a couple of exceptions, there appears to be no single version of the Stroop task in the literature that *simultaneously* (1) allows use of a set size of at least six (including six responses), which modern PC paradigms require in order to examine conflict-induced control independently from non-conflict processes; (2) allows data collection in other formats than the classic laboratory format, most notably in neuroimaging and online experiments; (3) uses non-arbitrary responses to targets, responses which are both not challenging for participants to learn and do not change the nature of the task in a potentially important way. Although, for example, manual color-word and picture-word Stroop-like tasks meet the first and second criteria, they do not meet the third. Similarly, the vocal versions of those tasks (i.e., proper Stroop tasks) meet the first and third criteria, but, in most cases, not the second. One exception is the Dual-Mechanisms of Control project, a project in which a vocal color-word Stroop task designed to examine conflict-induced control independently from non-conflict processes has been included in a task battery delivered online (Tang et al., 2023) and in neuroimaging sessions (Braver et al., 2021). However, as the authors of the project report, the online experiment was conducted with proprietary software, and in both the online and the neuroimaging batteries, the Stroop task was one of the tasks most impacted by data loss. The only other exception is represented by color-word Stroop tasks requiring participants to respond manually to colors, but to do so by *typing* the color name or its initial on a standard keyboard (i.e., a non-arbitrary response) rather than by pressing an arbitrary key (Crump et al., 2017; Logan & Zbrodoff, 1998). Those experiments are well suited for online experiments; however, they would not be for neuroimaging experiments that do not allow the use of a standard keyboard.

**Fig. 1:**
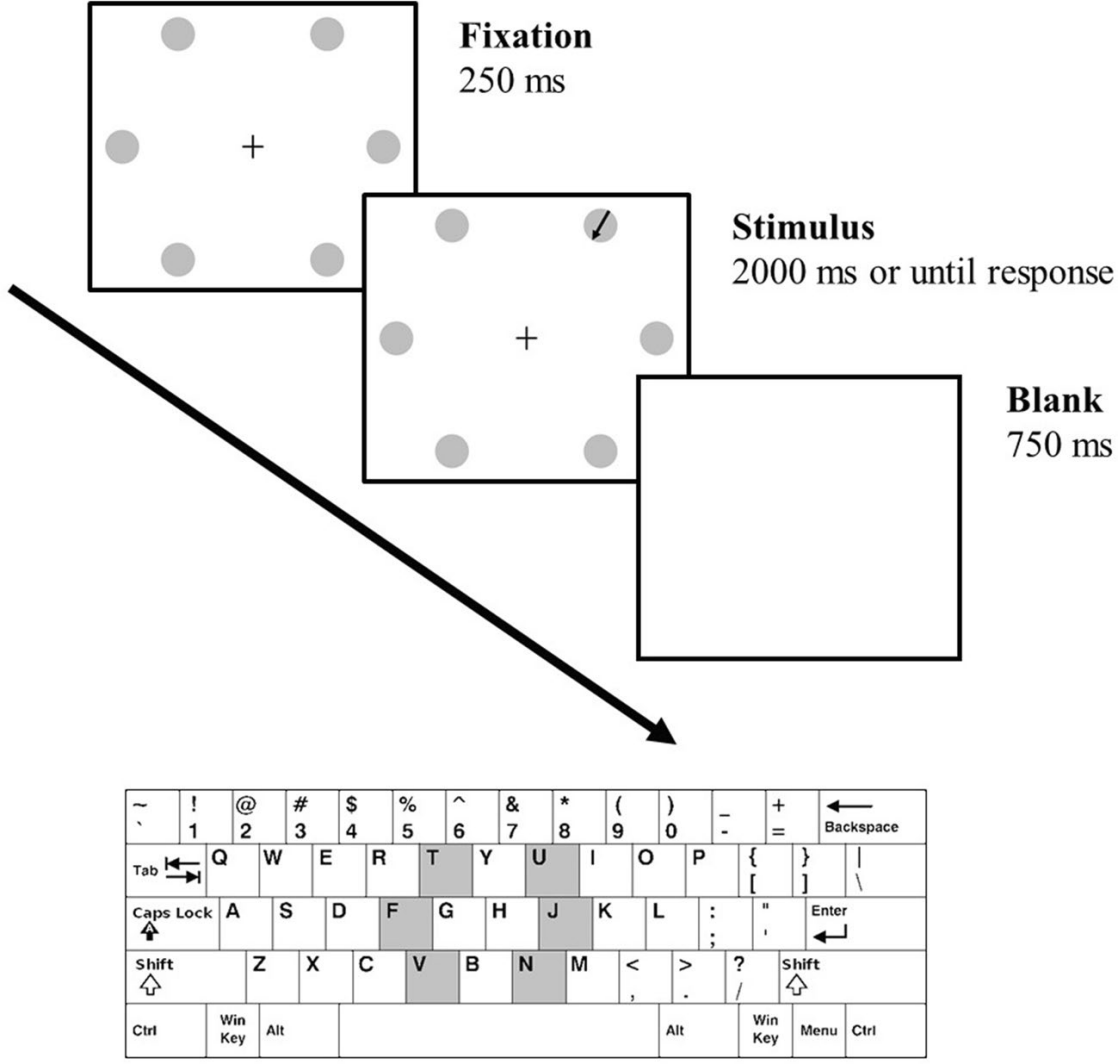
An illustration of the materials and procedure in the present research. The keys on the keyboard that are shaded in grey were used for responses. Participants were instructed to respond, as quickly and as accurately as possible, by pressing the button on the keyboard corresponding to the direction the arrow was pointing while ignoring the location in which the arrow was displayed. In this example, an incongruent item is presented in which a south-west-pointing arrow is displayed in the north-east location. A “south-west” response, indicated by pressing the spatially compatible V-key that occupies the south-west position in the response setup, would be required. The fact that the arrow is displayed in the north-east location, however, likely produces a tendency to respond with the spatially compatible U-key that occupies the north-east position in our response setup. The keyboard image was adapted from https://commons.wikimedia.org/wiki/File:KB_United_States.svg, distributed under the terms of CC BY-SA 3.0

Here, we present a spatial version of the Stroop task (henceforth, referred to as a “spatial Stroop task”) that also seems to meet all three criteria described above and, additionally, can be easily programmed with most experimental software and does not suffer from severe data loss issues. In this task, inspired by a similar task used by Puccioni and Vallesi (2012), participants are presented with six circles, or locations, in which an arrow can appear pointing in one of six possible directions. The participant’s task is to respond to the direction indicated by the arrow, ignoring its location, by pressing the corresponding key on the keyboard, with there being six keys designated for responses. Crucially, the positions of the keys used are spatially compatible with the arrows and locations used (for an illustration of the procedure, see Fig. 1).

Because in this task there are six targets (i.e., the arrows), six distractors (i.e., the locations), and six responses, this relatively large set size allows the implementation of the list-wide and item-specific PC paradigms that have been designed to control for non-conflict processes. Further, the manual nature of the response allows easy data collection not only in classic laboratory settings but also in other settings such as online experiments (the experiments reported below were, in fact, conducted online with freely distributed software). Finally, because the response keys are spatially compatible with the arrows and locations used, those stimulus-response associations should, on the one hand, be easy for participants to learn (including participants with reduced cognitive abilities), and on the other hand, create a situation in which the interference produced by the distractors involves at least some response conflict, paralleling the typical situation in color-word and picture-word Stroop tasks requiring vocal responses (Lu & Proctor, 2001). Note also that the non-verbal nature of the stimuli can be advantageous for research for which verbal stimuli would be undesirable, such as research examining proactive and reactive control in groups with different language abilities (e.g., Gullifer & Titone, 2021; Spinelli et al., 2022a).

Spatial versions of the Stroop task have already been used in combination with PC manipulations (e.g., Funes et al., 2010; Logan & Zbrodoff, 1979; Tafuro et al., 2020), but typically without controlling for non-conflict processes (or doing so in non-standard ways: Visalli et al., 2023). In order to illustrate the usefulness of this type of task, we present the results of two experiments in which the task was used to implement some of the designs that have been developed in order to examine proactive and reactive control independently from non-conflict processes in the list-wide (Experiment 1) and item-specific (Experiment 2) PC paradigms. Specifically, in Experiment 1 we used an inducer/diagnostic design to examine the list-wide PC effect, an effect associated with proactive control, and in Experiment 2 we used Spinelli and Lupker’s (2020) contingency-matching design to examine the item-specific PC effect, an effect associated with reactive control.

Note that this task is similar to, but not to be confused with, the Simon (1969) task (see, e.g., Lu & Proctor, 1995). As in the Simon task, the distractor is the location at which the stimulus appears. Unlike in the Simon task, however, the target requires a spatially compatible response (e.g., a “west” response when the arrow points west). In contrast, in the Simon task, the target is a color patch or a shape that is associated with a completely arbitrary response (e.g., a “west” response for a red color patch). This seemingly small difference has important implications. In Kornblum’s (1992) model, in particular, Simon tasks are classified as distinct “ensembles” (i.e., interference task types) from those of proper Stroop tasks. Specifically, Simon tasks are classified as type-3 ensembles, that is, ensembles in which representations for irrelevant stimulus components (e.g., left- and right-side locations) and responses (e.g., left- and right-side response buttons) overlap, but in which there is no overlap between either targets (e.g., red and green color patches) and responses or between targets and distractors. In contrast, as discussed in footnote 1, Stroop tasks are classified as type-8 ensembles, that is, ensembles involving all three types of overlap (i.e., distractor**–**response, target**–**response, and target**–**distractor). As recently argued by Viviani et al. (2023), spatial Stroop tasks (especially those which, like the present experiments, involve no linguistic material) rightfully belong to the type-8 ensemble (i.e., the Stroop ensemble), not to the type-3 ensemble (i.e., the Simon ensemble) because arrow directions, arrow locations, and response locations all have overlapping representations. Indeed, for this reason, according to Viviani et al., spatial Stroop tasks may be among the most promising ones in terms of carrying forward the Stroop legacy. The point, in any case, is that the type of task we used, albeit superficially similar to the Simon task, is best viewed as a Stroop task.

That said, our task and the manipulations we implemented with it can inform and inspire the literatures of other interference tasks, including but not limited to the Simon task. Indeed, interference tasks other than the color-word Stroop task are not exempt from the non-conflict processes that can affect performance in that task when using PC manipulations, and paradigms involving similar manipulations have been developed in an attempt to control for those processes in those tasks as well (e.g., picture-word: Bugg et al., 2011; flanker: Bugg & Gonthier, 2020; prime-probe: Schmidt, 2017). Along these lines, we have recently extended a list-wide PC manipulation used by Spinelli and Lupker (2023a) for a color-word Stroop task to other Stroop tasks as well as Stroop-like and Simon tasks. The experiments here presented could also be used as a starting point to implement similar manipulations in other interference tasks.

## Experiment 1

### Method

#### Participants

An a priori power analysis (the same analysis reported in Spinelli & Lupker, 2023a) was performed using G*Power 3.1 (Faul et al., 2009) to calculate the sample size needed for a power of .80 for obtaining a list-wide PC effect as large as the list-wide PC effects on diagnostic items reported by Bugg (2014) in her Experiments 1a and 2b in the latency data. Based on the smallest of those effect sizes (${\eta }_{p}^{2}$ = .190, reported for Bugg’s (2014) Experiment 1a), a minimum sample size of 38 participants would be needed. Fifty-five students at the University of Western Ontario participated in the experiment, which was conducted online, for course credit. After discarding too-fast, too-slow, and incorrect responses (see below), seven participants contributed fewer than 75% of their original observations. Those participants were removed from the analyses, leaving 48 participants (32 females and 16 males; five left-handed, 41 right-handed, and two ambidextrous; age 18–31 years). These criteria were determined a priori in line with previous work in our laboratory (Spinelli et al., 2020; Spinelli & Lupker, 2023a). All participants were native English speakers and had normal or corrected-to-normal vision.

#### Materials

An illustration of the materials and procedure used in this experiment is presented in Fig. 1. Six medium-grey circles centered on the vertices of an invisible regular hexagon were used to create distractor locations and black arrows pointing in one of six directions (north-east, east, south-east, south-west, west, and north-west, with a 60° angle between each successive direction) were used as targets. The hexagon, which had 222-pixel edges, was arranged so that the bottom and the top edges would be horizontal. As a result, three circles appeared on the right side of the figure and three on the left side. A regular hexagon was used so that the circles centered on the vertices of the hexagon would be equally distant from each other. On each trial, an arrow was presented inside one of the circles, with the length of the arrow corresponding to the diameter of the circle (58 pixels). A fixation symbol (“+”) was also displayed in the center of the hexagon. The figures for the stimuli were created with Powerpoint and had a 547-pixel width and a 480-pixel height.

The frequency of arrow-location combinations in one of the six counterbalancings of the experiment is represented in Tables 1 and 2 for the MC and MI list, respectively (in the following, this particular counterbalancing is used in all of our examples). Each arrow (e.g., the north-east-pointing arrow) was combined with two locations, the congruent location (e.g., the north-east location) and the incongruent location at the opposite vertex of the hexagon (e.g., the south-west location). The resulting stimuli were divided into two sets, with one set composed of four arrows and four locations (e.g., the north-east, south-west, south-east, and north-west arrows and locations) serving as the inducer set and another set composed of the remaining two arrows and two locations (e.g., the east and west arrows and locations) serving as the diagnostic set. The inducer set included more arrows and locations than the diagnostic set to allow for a strong manipulation of congruency proportion at the list level. Note that it is unlikely that using an inducer set with more arrows and locations than the diagnostic set would have any other impact because, from the participants’ perspective, there is no obvious separation between the two sets, nor were participants informed about the sets’ existence. Further, the stimuli in both sets were composed of arrows and locations that occurred a total of 32 times individually, making individual arrows and locations in the inducer set no more frequent than individual arrows and locations in the diagnostic set.

In the MC list, each location in the inducer set (e.g., the north-east location) appeared 30 times with the congruent arrow (e.g., the north-east-pointing location) and two times with its associated incongruent arrow (e.g., the south-west-pointing location). Overall, there were 120 congruent items and eight incongruent items in the inducer set in the MC list, an item-specific congruency proportion of 93.75%. In the MI list, the congruency proportion was reversed, with each location in the inducer set appearing two times with the congruent arrow and 30 times with its associated incongruent arrow. Overall, there were eight congruent items and 120 incongruent items in the inducer set in the MI list, an item-specific congruency proportion of 6.25%.

Each location in the diagnostic set (e.g., the east location), in contrast, appeared 16 times with the congruent arrow (e.g., the east-pointing arrow) and 16 times with its associated incongruent arrow (e.g., the west-pointing arrow) in both lists. Overall, there were 32 congruent items and 32 incongruent items in the diagnostic set in both lists, an item-specific congruency proportion of 50%. However, considering both inducer and diagnostic items, there were overall 152 congruent items and 40 incongruent items in the MC list (a list-wide congruency proportion of 79.17%) and 40 congruent items and 152 incongruent items in the MI list (a list-wide congruency proportion of 20.83%). In both lists, the inducer set and the diagnostic set were randomly intermixed. The assignment of the arrows and locations to inducer versus diagnostic items was counterbalanced across participants, thus controlling for potential processing differences among the arrow-location pairs that were assigned to the inducer and diagnostic sets. For example, processing for east- and west-pointing arrows, arrows for which a discrimination only along the horizontal axis is required, is likely faster than for the other arrows, which require a discrimination along both horizontal and vertical axes. However, east- and west-processing arrows were used as diagnostic stimuli only in the version of the experiment represented in Tables 1 and 2, with those arrows being used as inducer stimuli in other versions.

#### Procedure

An illustration of the materials and procedure is presented, as noted, in Fig. 1. Each trial began with a fixation figure in which the six circles, all empty, were displayed for 250 ms. Subsequently, an arrow was displayed in one of the circles for 2,000 ms or until the participant’s response. In both displays, a fixation symbol (“+”) was displayed in the center of the invisible hexagon. The hexagon itself was centered on the screen. Finally, there was a 750-ms blank screen between trials.

Participants were instructed to respond as quickly and as accurately as possible by pressing the button corresponding to the direction of the arrow while ignoring the arrow’s location. Specifically, they were instructed to press the U-key with the right middle finger for “north-east” responses, the J-key with the right index finger for “east” responses, the N-key with the right thumb for “south-east” responses, the V-key with the left thumb for “south-west” responses, the F-key with the left index finger for “west” responses, and the T-key with the left middle finger for “north-west” responses. Note that in keyboard layouts such as QWERTY, AZERTY, and QWETZ, these key positions are spatially compatible with the arrows and locations used. Because those layouts are by far the most common, we did not feel that there was a need to check the layout on participants’ computers. (However, in hindsight, it would probably be best for future users of this paradigm for remote testing to check with their participants that they are indeed using one of those keyboard layouts.) Participants were also invited to keep their elbows away from their chest in order for their hands to be tilted on the keyboard and more comfortable with the response arrangement.

The stimuli were presented against a white background in a full-screen browser window. The experiment was divided into two equal-sized blocks (192 trials per block) with a self-paced pause in the middle, one block being the MC list and the other being the MI list. The order in which the two lists were presented was counterbalanced across participants, and the order of trials within each list was randomized.

Initially, participants performed a practice session involving two blocks. The first block consisted of 30 trials in which a single circle was presented in the center of the screen. The circle was empty for 250 ms and then an arrow appeared inside it for 2,000 ms or until the participant’s response. The second block consisted of 48 trials, with the same materials and procedure as in the experimental session. However, in this practice block, unlike in the subsequent experimental blocks, there was no distinction between inducer and diagnostic items, because each location appeared four times with the congruent arrow and four times with its associated incongruent arrow (resulting in a congruency proportion of 50%). This somewhat longer practice session, compared to what is typical for vocal color-word and picture-word Stroop tasks, was included in order to allow participants to familiarize themselves with the stimulus-response mappings.

In line with previous work in our laboratory using the vocal color-word Stroop task (Spinelli & Lupker, 2020, 2021; Spinelli et al., 2020), no feedback was provided in the experimental session. Feedback, however, was provided in the practice session to facilitate learning of the stimulus-response mappings. In this session, after the stimulus display and before the blank screen, the feedback message “Correct” was displayed in green if the response made was correct, “Wrong” in red if the response was incorrect, and “No response,” also in red, if no response was made. All feedback messages were displayed in 36 pt Courier New Font for 500 ms. The experiment was run using the jsPsych (de Leeuw, 2015) JavaScript library.

### Results

Prior to all analyses, invalid trials due to responses faster than 300 ms or slower than 2,000 ms, the time limit (accounting for 1.1% of the data), were discarded.^3^ Prior to conducting the latency analyses, incorrect responses (accounting for 4.7% of the data) were also discarded. For this experiment and Experiment 2, all analyses were repeated using only trials following correct responses, and the pattern of results was virtually identical with respect to the crucial analyses. Also, for both experiments, the crucial analyses were repeated excluding participants (three in Experiment 1 and one in Experiment 2 for both latencies and error rates) for which at least one of the relevant condition means was associated with a studentized residual exceeding 3 in absolute value, suggesting a potentially strong influence of that condition mean on the results. Again, the pattern of results was virtually identical.

For both inducer and diagnostic items, a repeated-measures ANOVA was conducted on both latencies and errors with Congruency (Congruent vs. Incongruent) and List Type (Mostly congruent vs. Mostly incongruent) as within-subject factors. The analyses were repeated including the order in which participants received the lists (MC first vs. MI first) as an additional between-subject factor. These analyses revealed a practice effect in the response times (RTs; faster latencies in the second block than in the first block regardless of the type of list presented in the two blocks), but the pattern of results remained otherwise the same. In particular, the null three-way interaction between Congruency, List Type, and List Order that was obtained for both inducer items (*F*(1, 46) = .71, *MSE* = 2385, *p* = .405, ${\eta }_{p}^{2}$ = .015 for RTs, *F*(1, 46) < .01, *MSE* = .006, *p* = .948, ${\eta }_{p}^{2}$ < .001 for error rates) and diagnostic items (*F*(1, 46) = .16, *MSE* = 1299, *p* = .687, ${\eta }_{p}^{2}$ = .004 for RTs, *F*(1, 46) = .66, *MSE* = .002, *p* = .421, ${\eta }_{p}^{2}$ = .014 for error rates) indicates that the list-wide PC effect was not significantly smaller when the MI list was presented first than when the MC list was presented first, a pattern previously reported by Abrahamse et al. (2013). In any case, for simplicity, we report the analyses without the order factor.

In addition to traditional frequentist analyses, the evidence supporting the presence versus the absence of the list-wide PC effect, i.e., the Congruency by List Type interaction, was also quantified with Bayesian analyses comparing the model without that effect (interpreted as the null hypothesis *H*_0_) and the model with that effect (interpreted as the alternative hypothesis *H*_1_) in JASP version 0.16.41 (JASP Team, 2022) using the default settings. The result of this comparison is reported as *BF*_10_, with *BF*_10_ > 1 suggesting evidence in support of *H*_1_ (i.e., the presence of the effect), and *BF*_10_ < 1 suggesting evidence in support of *H*_0_ (i.e., the absence of the effect) (*BF*_10_ = 1 would suggest equal evidence for the two hypotheses).

Separate analyses were conducted for inducer and diagnostic items, paralleling previous research using the inducer/diagnostic design (e.g., Bugg, 2014; Bugg et al., 2008). Note that the analysis playing the crucial role in demonstrating conflict-induced control is that involving diagnostic items, whereas the analysis involving inducer items serves more as a manipulation check (i.e., because any of a number of processes can produce a PC effect for those items, it follows that that analysis must produce one, as it typically does, in order for the manipulation to be deemed minimally successful). In addition, to gain some insight into the psychometric properties of our manipulation, we conducted a reliability analysis of the list-wide PC effect produced by the diagnostic items (i.e., the crucial effect) by computing Spearman-Brown corrected split-half reliabilities using Parsons’ (2021) split-half package, version 0.8.2 in R version 4.2.2 (R Core Team, 2022), with random assignment to the two halves over 5,000 iterations.^4^

The mean RTs and error rates for the inducer and diagnostic items are presented in Tables 3 and 4, respectively. Skewness and kurtosis values for all of the conditions for both latencies and error rates, calculated using Komsta and Novomestky’s (2022) moments package, version 0.14.1 in R, are presented in Table 5. For this and the following experiment, the raw data, JASP files, and study materials are available via the Open Science Framework at https://osf.io/6v2p9/. Neither experiment was preregistered.

#### Inducer items

##### Response times (RTs)

There was a main effect of Congruency, *F*(1, 47) = 62.76, *MSE* = 3931, *p* < .001, ${\eta }_{p}^{2}$ = .572, indicating overall faster responses to congruent than incongruent items, but no main effect of List Type, *F*(1, 47) < .01, *MSE* = 7030, *p* = .973, ${\eta }_{p}^{2}$ < .001. However, List Type interacted with Congruency, *F*(1, 47) = 305.83, *MSE* = 2370, *p* < .001, ${\eta }_{p}^{2}$ = .867, *BF*_10_ = 5.10*10^28^ ± 2.78%. The interaction reflected the fact that the congruency effect was not just smaller in the MI list than in the MC list, the typical pattern of the list-wide PC effect – the congruency effect in the MI list was *reversed*, with responses to incongruent items being 51-ms *faster* than responses to congruent items (a significant difference, *t*(47) = 5.28, *p* < .001, ${\eta }_{p}^{2}$ = .372).

##### Error rates

There were main effects of Congruency, *F*(1, 47) = 34.83, *MSE* = .012, *p* < .001, ${\eta }_{p}^{2}$ = .426, with congruent items eliciting fewer errors than incongruent items, and List Type, *F*(1, 47) = 17.68, *MSE* = .008, *p* < .001, ${\eta }_{p}^{2}$ = .273, with the MI list eliciting fewer errors than the MC list overall. Congruency and List type interacted as well, *F*(1, 47) = 51.90, *MSE* = .006, *p* < .001, ${\eta }_{p}^{2}$ = .525, *BF*_10_ = 6.85*10^8^ ± 3.18%, indicating that the congruency effect was larger in the MC list (17.56%) than in the MI list (1.07%), the typical pattern of the list-wide PC effect. Although the congruency effect in the MI list was not reversed in this case, it was not statistically different from zero either, *t*(47) = -.86, *p* = .396, ${\eta }_{p}^{2}$ = .015.

#### Diagnostic items

##### RTs

There was a main effect of Congruency, *F*(1, 47) = 69.66, *MSE* = 3635, *p* < .001, ${\eta }_{p}^{2}$ = .597, indicating faster responses to congruent than incongruent items, but no main effect of List Type, *F*(1, 47) = .05, *MSE* = 6213, *p* = .829, ${\eta }_{p}^{2}$ < .001. Importantly, Congruency and List Type interacted, *F*(1, 47) = 98.70, *MSE* = 1276, *p* < .001, ${\eta }_{p}^{2}$ = .677, *BF*_10_ = 6.78*10^10^ ± 3.39%. The congruency effect was larger in the MC list (124 ms) than in the MI list (22 ms), the typical list-wide PC effect pattern. Note that the 22-ms congruency effect in the MI list was significant, *t*(47) = -2.10, *p* = .041, ${\eta }_{p}^{2}$ = .086.

##### Error rates

There were main effects of Congruency, *F*(1, 47) = 52.25, *MSE* = .006, *p* < .001, ${\eta }_{p}^{2}$ = .526, with congruent items eliciting fewer errors than incongruent items, and List Type, *F*(1, 47) = 10.56, *MSE* = .004, *p* = .002, ${\eta }_{p}^{2}$ = .183, with the MI list eliciting fewer errors than the MC list overall. Congruency and List type interacted, *F*(1, 47) = 23.95, *MSE* = .002, *p* < .001, ${\eta }_{p}^{2}$ = .338, *BF*_10_ = 2918.95 ±10.39%, indicating that the congruency effect was larger in the MC list (11.29%) than in the MI list (4.57%), the typical list-wide PC effect pattern. Note that, in these data as well, the 4.57% congruency effect in the MI list was significant, *t*(47) = -5.41, *p* < .001, ${\eta }_{p}^{2}$ = .383.

#### Reliability analysis

**Fig. 2:**
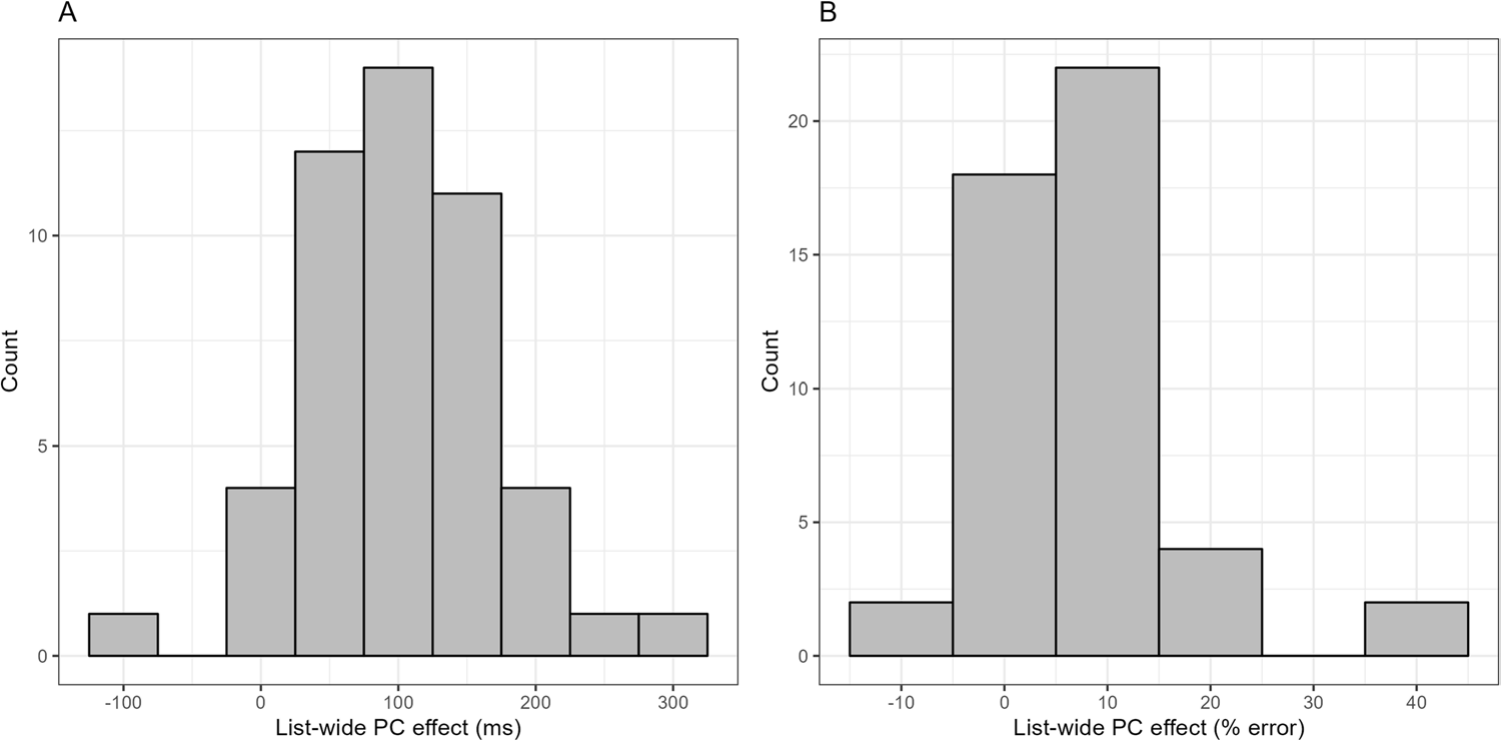
Histogram of the list-wide Proportion-Congruent (PC) effects for the diagnostic items in Experiment 1. The list-wide PC effect is calculated by subtracting, for both latencies and error rates, the participant’s congruency effect in the Mostly-Incongruent (MI) list from the participant’s congruency effect in the Mostly-Congruent (MC) list

A histogram of the list-wide PC effects (calculated by subtracting, for both latencies and error rates, the participant’s congruency effect in the MI list from the participant’s congruency effect in the MC list) for the diagnostic items is presented in Fig. 2. For the latencies (Fig. 2A), skewness was .26, kurtosis was 3.84, and 45 participants (out of 48, i.e., 93.75%) showed a positive effect (i.e., an effect in the expected direction). For the error rates (Fig. 2B), skewness was 1.64, kurtosis was 6.91, and 33 participants (i.e., 68.75%) showed a positive effect. Despite the general robustness of the list-wide PC effect, the Spearman-Brown corrected split-half reliabilities were only *r*_SB_ = .12, 95% CI [-.38, 0.52] for the latencies, and *r*_SB_ = .27, 95% CI [-.12, 0.57] for the error rates.

### Discussion

Not surprisingly, both RTs and error rates showed a list-wide PC effect for inducer items. Interestingly, this list-wide PC effect reflected a complete elimination of the congruency effect in the MI list for the error rates and, for the latencies, a reversal of the effect. Note that this reversed congruency effect is not new in the literature (indeed, in one of the first ever list-wide PC manipulations, Logan & Zbrodoff (1979) reported a reversed congruency effect in the MI list in a spatial Stroop task similar to ours, albeit with a much simpler design; for similar evidence in the Simon task, see Borgmann et al., 2007; for evidence from a Stroop task with an inducer/diagnostic design, see Blais & Bunge, 2010). However, it is not an effect that can be explained by standard control accounts (e.g., Botvinick et al., 2001) because those accounts predict a reduction or, at most, an elimination of the processing cost associated with conflict (i.e., the congruency effect) in situations in which conflict is more frequent. That is, conflicting stimuli should never become easier to process than non-conflicting stimuli according to standard control accounts (but for an alternative control account that would be able to explain reversed congruency effects, see Weissman et al., 2015).

The most likely explanation for the reversed congruency effect is that the effect is the result of a non-conflict factor that, for inducer items, is confounded with the congruency proportion manipulation. For example, for those items, contingency learning in the MI list might have facilitated responses to incongruent stimuli, stimuli that were high-contingency for inducer items, to the point that those stimuli were responded to faster than congruent stimuli, stimuli that were low-contingency for inducer items. Alternatively, the reversal might result from the fact that each individual incongruent stimulus was much more frequent (30 occurrences) than each individual congruent stimulus (two occurrences) for inducer items, creating a strong repetition-priming effect for the incongruent stimuli such that they then produced shorter latencies than the congruent stimuli. Although the fact that inducer items produced a list-wide PC effect is hardly surprising, the fact that the pattern of the effect involved a reversal of the congruency effect in the MI list appears to be a nice demonstration of the strong impact that non-conflict processes can have in PC paradigms and why it is important to control for that impact.

More importantly for present purposes, both RTs and error rates showed a regular list-wide PC effect for *diagnostic* items, with a larger congruency effect when those items appeared in the MC compared to the MI list, with those items in the MI list still showing a typical, albeit reduced, congruency effect (incongruent harder to process than congruent). Because those items were identical in the two lists, non-conflict processes should have had a similar impact, if they had any impact at all, on them in the two lists. Therefore, the observed effects must be due to the nature of the list that those items appeared in, presumably reflecting an item-nonspecific process of proactive control increasing selective attention to target information in the MI list compared to the MC list (but for alternative explanations, see the *General discussion*). Note, further, that the effect sizes for the PC effects (${\eta }_{p}^{2}$ = .677 and ${\eta }_{p}^{2}$ = .338 for RTs and error rates, respectively) were quite large in comparison to what is typically reported for diagnostic items (e.g., Spinelli & Lupker, 2023a, reported effect sizes ranging from ${\eta }_{p}^{2}$ = .276 to ${\eta }_{p}^{2}$ = .361 for RTs and from ${\eta }_{p}^{2}$ = .056 to ${\eta }_{p}^{2}$ = .118 for error rates in their color-word Stroop experiments), suggesting that our task was particularly effective at inducing a proactive control modulation.

While the list-wide PC effect for the diagnostic items was robust, it was associated with poor reliability. Part of the reason for this poor reliability is that the list-wide PC effect is a difference score (more precisely, a difference of difference scores), a type of score that inevitably has lower reliability than its components (Draheim et al., 2019; Rodebaugh et al., 2016). In general, this type of situation is yet another example of the “reliability paradox” affecting many classic tasks in cognitive psychology, tasks that produce effects that are, in most cases, robust, but do not produce high reliability coefficients (Hedge et al., 2018).

## Experiment 2

In Experiment 2, we tested the effectiveness of the spatial Stroop task used in Experiment 1 at engaging item-specific, reactive control by examining data from the item-specific PC paradigm. In order to examine the impact of reactive control independently from the impact of non-conflict processes, we used Spinelli and Lupker’s (2020) design. In that design, implemented in the color-word Stroop task, Spinelli and Lupker constructed the MC set by presenting each of three colors (e.g., red, yellow, and black) with its congruent word (e.g., RED for the color red, a high-contingency stimulus) more often than with the other two (incongruent) words used in that set (e.g., YELLOW and BLACK for the color red, both low-contingency stimuli). Similarly, the MI set was constructed by presenting each of three different colors (e.g., blue, green, and white) more often in one of the other (incongruent) words used in that set (e.g., GREEN for the color white, a high-contingency stimulus) than with either its congruent word or the third (incongruent) word (e.g., WHITE and BLUE, respectively, for the color white, both low-contingency stimuli). The advantage of this design is that it produces two types of low-contingency incongruent stimuli: MC stimuli (e.g., YELLOW in red and BLACK in red) and MI stimuli (e.g., BLUE in white). Because these stimuli are matched on contingency learning and, further, are presented with the same individual frequency in the experiment, they are only differentiated by the MC versus MI nature (i.e., red, yellow, and black words and colors – the MC stimuli – usually indicate that the stimulus is a congruent stimulus, whereas blue, green, and white words and colors – the MI stimuli – usually indicate that the stimulus is an incongruent stimulus). Therefore, the fact that Spinelli and Lupker observed longer latencies for the MC incongruent stimuli than for the MI incongruent stimuli in that particular contrast may only be attributed to selective attention to target information being increased in response to the latter stimuli, a reactive-control process.

In the present experiment, we adapted Spinelli and Lupker’s (2020) design to the spatial Stroop task used in the present Experiment 1. Although the nature of the stimuli in that task is different from those in the color-word version of the task, the logic was similar: Because the stimuli being compared in the crucial contrast (the infrequent incongruent stimuli in the MC and MI sets) would be matched on contingency learning and individual stimulus frequency but not on item-specific conflict frequency (i.e., the conflict frequency associated with the particular targets and/or a particular distractors in each set), any difference between those stimuli would be the result of a reactive-control process. Specifically, increased latencies and/or error rates for the MC incongruent stimuli compared to those MI incongruent stimuli in the contrast would be consistent with the idea that selective attention to target information is reactively increased for the latter stimuli.

### Method

#### Participants

An a priori power analysis was performed using G*Power 3.1 (Faul et al., 2009) to calculate the sample size needed for a power of .80 for obtaining an effect as large as the effect reported by Spinelli and Lupker (2020) for the contrast between MC incongruent stimuli and contingency-matched MI incongruent stimuli in latencies, ${\eta }_{p}^{2}$ = .088. This analysis revealed that a minimum sample size of 85 participants would be needed. 104 students at the University of Western Ontario participated in this experiment, which was conducted online, for course credit. After discarding too-fast, too-slow, and incorrect responses (see below), eight participants contributed fewer than 75% of their original observations. Those participants were removed from the analyses, leaving 96 participants (58 females and 38 males; 11 left-handed, 84 right-handed, and one ambidextrous; age 18–24 years). All participants were native English speakers and had normal or corrected-to-normal vision.

#### Materials

The materials were the same as in Experiment 1. What changed was how the arrows and the locations were combined. The frequency of arrow-location combinations in one of the four counterbalancings of the experiment was modelled after Spinelli and Lupker (2020) and is represented in Table 6 (in the following, this particular counterbalancing will be used in all of our examples). In this experiment, each arrow (e.g., the north-east-pointing arrow) was combined with three locations, the congruent location (e.g., the north-east location) and the two incongruent locations on the same side of the hexagon (left or right) as the congruent location (e.g., the east and the south-east locations). The resulting two sets of stimuli (one for the left side, the other for the right side) were manipulated either as an MC set or as an MI set. In the MC set, each location appeared with its congruent arrow 48 times and with each of the two incongruent arrows eight times, resulting in an item-specific (i.e., location-specific and arrow-specific) congruency proportion of 75%. Similarly, in the MI set, each location appeared with one incongruent arrow 48 times and with both the other incongruent arrow and the congruent arrow eight times, resulting in an item-specific congruency proportion of 12.5%.

**Table 1:**
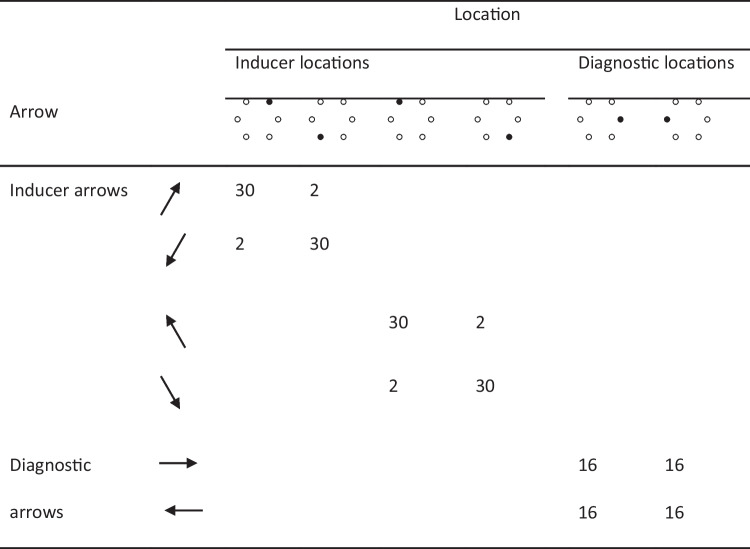
Template for the frequency of arrow-location combinations in the Mostly-Congruent (MC) list in Experiment 1

**Table 2:**
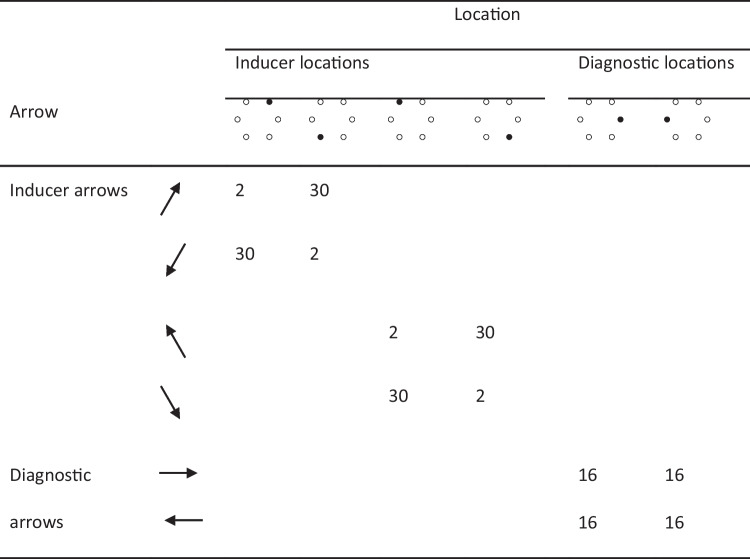
Template for the frequency of arrow-location combinations in the Mostly-Incongruent (MI) list in Experiment 1

**Table 3:** Mean response times (RTs) and percentage error rates (and corresponding standard deviations) for inducer items in Experiment 1

	RTs		Error rates	
Item type	MC list	MI list	MC list	MI list
Congruent	576 (100)	699 (122)	1.57 (2.04)	4.35 (8.06)
Incongruent	770 (123)	648 (113)	19.13 (17.23)	5.42 (6.78)
Congruency effect	194	-51	17.56	1.07

**Table 4:** Mean response times (RTs) and percentage error rates (and corresponding standard deviations) for diagnostic items in Experiment 1

	RTs		Error rates	
Congruency	MC list	MI list	MC list	MI list
Congruent	589 (108)	643 (127)	2.21 (4.36)	2.46 (4.11)
Incongruent	713 (132)	665 (147)	13.50 (13.14)	7.03 (7.57)
Congruency effect	124	22	11.29	4.57

**Table 5:** Skewness and kurtosis values for response times (RTs) and percentage error rates for inducer and diagnostic items in Experiment 1

Dependent variable	Item type	List type	Congruency	Skewness	Kurtosis
RTs	Inducer	MC list	Congruent	1.94	8.29
			Incongruent	.48	2.30
		MI list	Congruent	.81	4.30
			Incongruent	1.40	5.13
	Diagnostic	MC list	Congruent	1.79	8.71
			Incongruent	1.16	5.12
		MI list	Congruent	1.77	7.74
			Incongruent	1.68	6.70
Error rates	Inducer	MC list	Congruent	2.30	9.10
			Incongruent	0.66	2.67
		MI list	Congruent	2.55	11.70
			Incongruent	3.43	16.10
	Diagnostic	MC list	Congruent	2.73	9.96
			Incongruent	2.89	13.80
		MI list	Congruent	1.77	5.26
			Incongruent	2.31	9.64

**Table 6:**
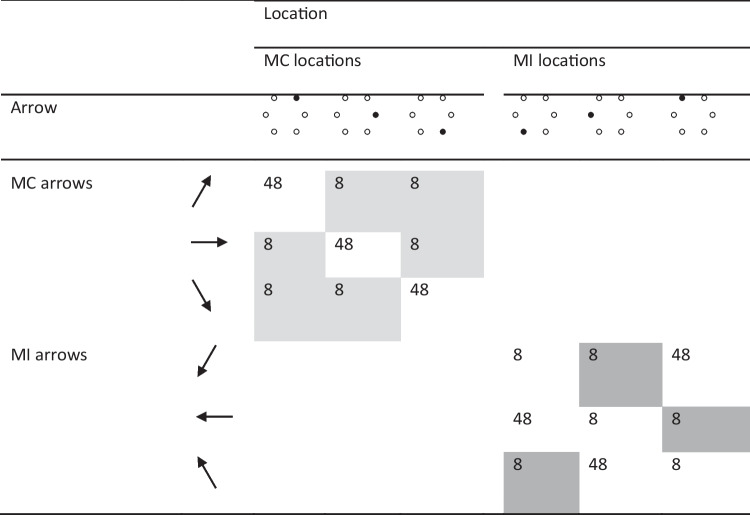
Template for the frequency of arrow-location combinations in Experiment 2

The incongruent items shaded in light grey are the Mostly-Congruent (MC) incongruent items, those shaded in dark grey are the Mostly-Incongruent (MI) incongruent items matched on contingency learning and individual stimulus frequency with the MC incongruent items

**Table 7:** Mean response times (RTs) and percentage error rates (and corresponding standard deviations) for all of the conditions involved in Experiment 2

	RTs		Error rates	
Congruency	MC items	MI items	MC items	MI items
Congruent	589 (101)	616 (101)	1.56 (1.75)	1.27 (2.54)
Low-contingency incongruent	752 (135)	749 (128)	13.52 (9.70)	9.41 (8.33)
High-contingency incongruent		704 (119)		7.37 (7.48)
Incongruent (collapsed)	752 (135)	710 (119)	13.52 (9.70)	7.66 (7.24)
Congruency effect (incongruent collapsed − congruent)	163	94	11.96	6.39

Note that the arrows and locations used for MC and MI sets were not permitted to overlap in order to avoid creating stimuli with an ambiguous congruency proportion (e.g., an MC arrow appearing in an MI location; see Spinelli & Lupker, 2020). Further, the arrows and locations used for each set were not permitted to cross sides (i.e., left-side arrows appeared only in left-side locations and right-side arrows appeared only in right-side locations) because responses to left-side arrows (i.e., north-west-, west-, and south-west-pointing arrows) were done with one hand (the left hand) and responses to right-side arrows (i.e., north-east-, east-, and south-east-pointing arrows) were done with the other hand (the right hand). Because conflict-induced control processes sometimes do not generalize across responding hands (e.g., Kim & Cho, 2014; Lim & Cho, 2018), we decided to maintain, for each participant, each of the set types (MC vs. MI) on one of the responding hands.

An implication of this design is that, should a reactive-control effect unconfounded from non-conflict processes emerge when contrasting MC and MI sets, that effect might not be “item-specific” in the sense that it is triggered by recognition of individual stimulus components (e.g., a south-west-pointing arrow as opposed to a west-pointing one). The effect might be “side-specific” because recognition of the side (left vs. right) of the stimulus might be sufficient to trigger reactive control (similar to the location-specific PC effects reported for context-specific PC manipulations in color-word Stroop tasks: Crump et al., 2006). For example, the presentation of an MI stimulus such as the south-west-pointing arrow displayed in the west location might cause a selective-attention increase not only because that particular arrow or that particular location is associated with conflict, but also because “left” stimuli are generally associated with conflict in this experiment. However, even if the effect is “side-specific” and not “item-specific”, it would still be a reactive-control effect since participants in this experiment do not know and, hence, cannot prepare for the side of the upcoming stimulus. Since our interest lies in examining reactive control, the exact nature of this form of control in the present experiment is not of primary importance.

Note, finally, that the MI set was designed in a symmetric fashion compared to the MC set (with each location appearing 48 times with an arrow and eight times with the other arrows in the set) in order for contingency learning and individual stimulus frequency to be perfectly matched in the crucial contrast between the two types of incongruent stimuli (i.e., those in the MI vs. the MC sets). That is, those stimuli were designed in such a way that they would only differ in item-specific conflict frequency: Each incongruent stimulus in the MC set was a low-contingency stimulus appearing eight times in the experiment (e.g., the east-pointing arrow appearing in the north-east location, shaded in light grey in Table 6), and each incongruent stimulus in the MI set used in the crucial contrast was also a low-contingency stimulus appearing eight times in the experiment (e.g., the north-west-pointing arrow appearing in the south-west location, shaded in dark grey in Table 6). In the following, for simplicity, we call this contrast (and associated effects) the “reactive-control” contrast (effects) based on the assumption that, should a difference emerge in this contrast, it would have to be attributed to reactive control and no other process. Importantly, this contrast is only based on incongruent stimuli because, for congruent stimuli, it is impossible to fully control for non-conflict processes in an item-specific PC manipulation. Overall, there were 384 items (168 congruent and 216 incongruent). The assignment of each set to the MC or the MI condition was counterbalanced across participants. The specific incongruent arrow serving as the high-contingency arrow for locations in the MI set was also counterbalanced across participants.

#### Procedure

The procedure was the same as in Experiment 1 except that the composition of the second practice block mirrored that of the upcoming experimental blocks, as is common in item-specific PC paradigms (e.g., Spinelli & Lupker, 2020; Spinelli et al., 2020, 2022b). For example, in the second practice block for the counterbalancing presented in Table 6, the north-east location appeared six times with its congruent arrow, once with the east-pointing arrow, and once with the south-east-pointing arrow, and so on for the other locations and arrows. As in Experiment 1, trials were randomized and there were two experimental blocks with a self-paced pause in the middle; however, there was no difference in the nature of the stimuli in the two blocks.

### Results

Prior to all analyses, invalid trials due to responses faster than 300 ms or slower than 2,000 ms, the time limit (accounting for 1.6% of the data), were discarded. Prior to the RT analyses, incorrect responses (accounting for 5.6% of the data) were also discarded. The design used in this experiment allowed us to conduct three analyses: a classic item-specific PC analysis contrasting overall congruency effects for MC items versus MI items, a contingency-learning analysis contrasting MI incongruent stimuli matched on item-specific (i.e., arrow- and location-specific) conflict frequency, and a reactive-control analysis contrasting incongruent stimuli belonging to the MC set versus the MI set but matched on contingency learning and individual stimulus frequency.

The classic item-specific PC analysis was conducted using a repeated-measures ANOVA with Congruency (Congruent vs. Incongruent) and Item Type (Mostly congruent vs. Mostly incongruent) as within-subject factors, with no distinction being made between the low-contingency and the high-contingency incongruent items in the MI set (the data from all incongruent items in the MI set were collapsed). This analysis serves as a manipulation check (the same role played by the analysis of inducer items in Experiment 1), that is, it provides a demonstration that the task produces the expected item-specific PC effect in a situation in which a number of processes (including non-conflict ones) could produce it, the most basic goal for a successful item-specific PC manipulation.

The contingency-learning analysis was conducted by contrasting low-contingency MI incongruent stimuli (e.g., in the counterbalancing presented in Table 6, the north-west-pointing arrow appearing in the south-west location) and high-contingency MI incongruent stimuli (e.g., the west-pointing arrow appearing in the south-west location). Those types of stimuli were matched on item-specific conflict frequency because they both belonged to the MI set but differed on contingency learning as well as individual stimulus frequency (as each low-contingency stimulus appeared eight times in the experiment whereas each high-contingency stimulus appeared 48 times). Thus, a difference between them, in particular, an advantage for the high-contingency stimuli compared to the low-contingency stimuli, would have to be interpreted as the effect of contingency learning and/or repetition-priming processes. This idea was examined using a one-tailed *t*-test (using both a frequentist and a Bayesian approach) reflecting the alternative hypothesis that high-contingency stimuli would elicit lower RTs and error rates than the matched low-contingency stimuli.

Finally, the reactive-control analysis was conducted by contrasting incongruent stimuli belonging to the MC set versus the MI set but matched on contingency learning and individual stimulus frequency. This contrast was conducted with a one-tailed *t*-test (using both a frequentist and a Bayesian approach) reflecting the alternative hypothesis that the former stimuli would elicit higher RTs and error rates than the latter (for similar analyses, see Bugg & Hutchison, 2013; Spinelli et al., 2022b). As noted, this contrast is crucial because, should a difference emerge, it would have to be attributed to reactive control and no other process. Further, for this contrast, similar to what we did for the crucial contrast in Experiment 1 (i.e., the list-wide PC effect for diagnostic items), we also conducted a reliability analysis. Note that the reason for this contrast to involve incongruent stimuli only is that congruent stimuli in MC and MI conditions were not matched on non-conflict processes in the present manipulation: In the MC condition, they were high-contingency and appeared with a high individual stimulus frequency (i.e., 48 times each), whereas in the MI condition, they were low-contingency and appeared with a low individual stimulus frequency (i.e., eight times each). Therefore, the contrast between those stimuli cannot be used to provide unambiguous evidence for reactive control. Instead, we focused on the incongruent stimuli matched on non-conflict processes only, and invite future users of this manipulation to do so as well. The mean RTs and error rates for all of the conditions involved in Experiment 2 are presented in Table 7 . In that table, in addition to the congruent, low-contingency incongruent, and high-contingency incongruent conditions, we also report the data for all incongruent conditions collapsed and for the congruency effects obtained by contrasting that collapsed incongruent condition with the congruent condition. The classic item-specific PC analysis was based on that contrast. The contrasts on which the contingency-learning and reactive-control analyses were based are reported in more detail in Tables 8 and 9, respectively. Skewness and kurtosis values for all of the conditions for both latencies and error rates are presented in Table 10.

**Table 8:** Mean response times (RTs) and percentage error rates (and corresponding standard errors) for low-contingency and high-contingency incongruent stimuli in the Mostly-Incongruent (MI) condition in Experiment 2

Contingency	RTs	Error rates
Low	749 (128)	9.41 (8.33)
High	704 (119)	7.37 (7.48)
Contingency-learning effect	45	2.04

**Table 9:** Mean response times (RTs) and percentage error rates (and corresponding standard deviations) for incongruent stimuli in the Mostly-Congruent (MC) condition and matched incongruent stimuli in the Mostly-Incongruent (MI) condition in Experiment 2

Condition	RTs	Error rates
MC	752 (135)	13.52 (9.70)
MI	749 (128)	9.41 (8.33)
Reactive-control effect	3	4.11

**Table 10:** Skewness and kurtosis values for response times (RTs) and percentage error rates for all the conditions in Experiment 2

Dependent variable	Item type	Congruency	Contingency	Skewness	Kurtosis
RTs	MC	Congruent	High	.73	3.67
		Incongruent	Low	.64	3.63
	MI	Congruent	Low	.29	2.39
		Incongruent	High	.17	2.36
		Incongruent	Low	.32	2.60
Error rates	MC	Congruent	High	1.75	6.72
		Incongruent	Low	.96	3.22
	MI	Congruent	Low	2.45	10.10
		Incongruent	High	2.09	7.61
		Incongruent	Low	.88	3.42

#### Classic item-specific Proportion-Congruent (PC) analysis

##### RTs

There was a main effect of Congruency, *F*(1, 95) = 763.53, *MSE* = 2083, *p* < .001, ${\eta }_{p}^{2}$ = .889, indicating faster responses to congruent than incongruent items, and a marginal effect of Item Type, *F*(1, 95) = 2.77, *MSE* = 2069, *p* = .099, ${\eta }_{p}^{2}$ = .028. Congruency and Item Type interacted, *F*(1, 95) = 176.68, *MSE* = 630, *p* < .001, ${\eta }_{p}^{2}$ = .650, *BF*_10_ = 7.65*10^20^ ± 7.78%, as the congruency effect was larger for MC items (163 ms) than for MI items (94 ms), the typical pattern for the item-specific PC effect.

##### Error rates

There were main effects of Congruency, *F*(1, 95) = 141.15, *MSE* = .006, *p* < .001, ${\eta }_{p}^{2}$ = .598, with congruent items eliciting fewer errors than incongruent items, and Item Type, *F*(1, 95) = 90.77, *MSE* = .001, *p* < .001, ${\eta }_{p}^{2}$ = .489, with MI items eliciting fewer errors than the MC items overall. Congruency and Item Type interacted in this case as well, *F*(1, 95) = 75.82, *MSE* = .001, *p* < .001, ${\eta }_{p}^{2}$ = .444, *BF*_10_ = 4.58 ± 13.32%, indicating that the congruency effect was larger for MC items (11.96%) than for MI items (6.39%), the typical pattern for the item-specific PC effect.

#### Contingency-learning analysis

##### RTs

The high-contingency incongruent stimuli in the MI set (704 ms) were significantly faster than the low-contingency incongruent stimuli in that set (749 ms), *t*(95) = -10.56, *p* < .001, ${\eta }_{p}^{2}$ = .540, *BF*_-0_ = 1.10*10^15^ (note that the minus in the subscript of the Bayes factor denotes the directionality of the alternative hypothesis).

##### Error rates

The error rate for the high-contingency incongruent stimuli in the MI set (7.37%) was significantly lower than the error rate for the low-contingency incongruent stimuli in that set (9.41%), *t*(95) = -3.01, *p* = .002, ${\eta }_{p}^{2}$ = .087, *BF*_-0_ = 15.13.

#### Reactive-control analysis

##### RTs

The incongruent stimuli in the MC condition (752 ms) were only 3 ms slower than the matched incongruent stimuli in the MI condition (749 ms), a non-significant difference in a one-tailed *t*-test, *t*(95) = .43, *p* = .333, ${\eta }_{p}^{2}$ = .002, *BF*_+0_ = .16.

##### Error rates

The error rate was larger for the incongruent stimuli in the MC condition (13.52%) than for the matched incongruent stimuli in the MI condition (9.41%), a difference that was significant in a one-tailed *t*-test, *t*(95) = 5.60, *p* < .001, ${\eta }_{p}^{2}$ = .248, *BF*_+0_ = 1.24*10^5^.

#### Reliability analysis

**Fig. 3:**
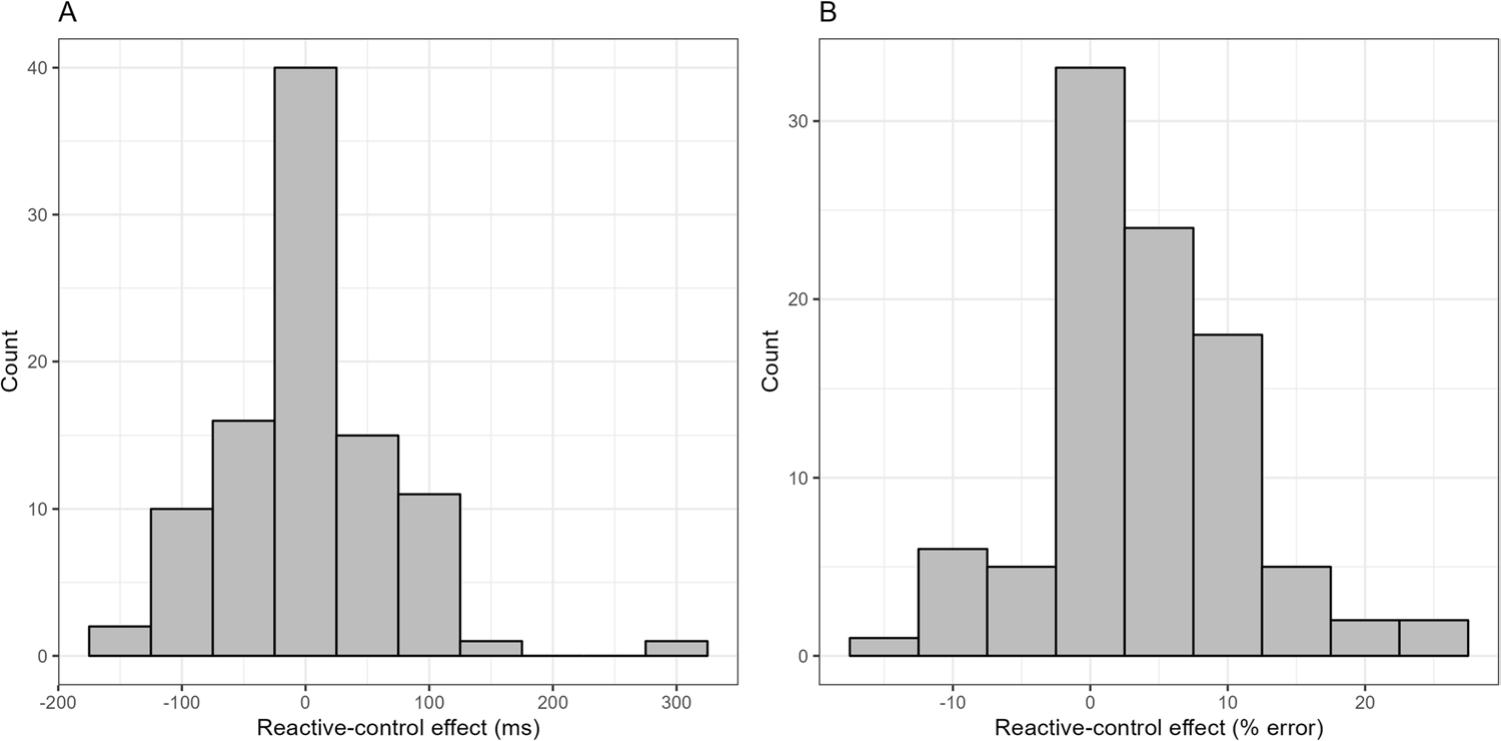
Histogram of the reactive-control effect in Experiment 2. The reactive-control effect is calculated by subtracting the participant’s mean latency (or error rate) for the low-contingency, incongruent, Mostly-Incongruent (MI) condition from the participant’s mean latency (or error rate) for the low-contingency, incongruent, Mostly-Congruent (MC) condition

A histogram of the reactive-control effects is presented in Fig. 3. For the latencies (Fig. 3A), skewness was .65, kurtosis was 6.06, and 54 participants (out of 96, i.e., 56.25%) showed a positive effect (i.e., an effect in the expected direction). For the error rates (Fig. 3B), skewness was .27, kurtosis was 3.54, and 67 participants (i.e., 69.79%) showed a positive effect. Overall, the effect was not as robust as the list-wide PC effect for diagnostic items in Experiment 1. Further, the Spearman-Brown-corrected split-half reliabilities were only *r*_SB_ = .39, 95% CI [0.19, 0.57] for the latencies, and *r*_SB_ = -.09, 95% CI [-.34, 0.20] for the error rates.

### Discussion

The analyses conducted for Experiment 2 produced the following results. First, when using a classic item-specific PC analysis contrasting congruency effects for MC and MI items (with no distinctions being made between the different types of incongruent stimuli used in the design), we found that the present experiment, as with most item-specific PC experiments, was successful at producing a regular item-specific PC effect in both RTs and error rates, with a larger congruency effect for MC items than for MI items. Second, when contrasting stimuli matched on item-specific conflict frequency but differing on contingency learning as well as individual stimulus frequency, responses to high-contingency incongruent stimuli, that is, stimuli that required their typical response and were repeated more times in the experiment, were faster and more accurate than responses to low-contingency incongruent stimuli, that is, stimuli that required an atypical response for them and were repeated fewer times in the experiment. This effect may be interpretable as resulting from either a contingency-learning process (with locations being associated with their typical response) and/or a repetition-priming process (with stimuli repeated more times in the experiment being advantaged). In any case, the point is that, in item-specific PC manipulations as well as in list-wide PC manipulations, non-conflict processes do have an impact.

Finally and most importantly, although non-conflict processes did have an impact, Experiment 2 produced evidence for reactive control unconfounded with non-conflict processes, as incongruent stimuli matched on contingency learning and individual stimulus frequency elicited more errors when they belonged to the MC set, a stimulus set associated with infrequent conflict, than when they belonged to the MI set, a stimulus set associated with frequent conflict (a reactive-control effect). This result was not observed for RTs, however.

A possible, but speculative, interpretation of this pattern is that the primary impact of reactive control in the present task was to prevent the task goal from being neglected for MI stimuli as often as it is for MC stimuli (for similar explanations, see Kane & Engle, 2003; Spinelli et al., 2020). That is, when an MC stimulus is presented, the fact that selective attention is not inevitably increased may occasionally allow a response to the location instead of the direction of the arrow. Although this process would often result in a correct response for MC stimuli because most of those stimuli are congruent, it would result in an error for the rare incongruent stimuli in this condition on some occasions. However, on the other occasions (i.e., the occasions in which the task goal is correctly maintained), the direction of the arrow would be correctly identified without producing an increased latency.

In contrast, the selective-attention increase for MI stimuli would almost always avoid goal neglect when such a stimulus is presented. The reason is that, because most of those stimuli are incongruent, responding to the location instead of the direction of the arrow would result in an error in most cases. Thus, although latencies would be comparable for incongruent stimuli in this condition compared to the matched stimuli in the MC condition when the task goal is correctly maintained, there would be fewer episodes in which the goal is *not* maintained, resulting in lower error rates in this condition.

What might also have played some role in preventing a reactive-control effect from emerging in the latencies is the fact that the contingency-learning effect was relatively large in that dependent measure (45 ms and ${\eta }_{p}^{2}$ = .540). In particular, it was a bit larger than the 37-ms (and ${\eta }_{p}^{2}$ = .402) contingency-learning effect we reported for the original color-word Stroop experiment on which the present Experiment 2 was based (Spinelli & Lupker, 2020). In that color-word Stroop experiment, as noted, the presence of a contingency-learning effect did not prevent a reactive-control effect from also arising in the latencies. However, as we discuss more fully in the *General discussion* (section “Direct and indirect impacts of non-conflict processes”), there is an argument that contingency learning and reactive control may be competing with one another, with the former being prioritized when it can be used to minimize interference in the MI condition (Bugg et al., 2011; see also Bugg & Hutchison, 2013), as was the case for the present Experiment 2. Based on these considerations, it is possible that in the present Experiment 2, contingency learning was strong enough to eliminate a large portion of the conflict cost produced by incongruent distractors in the MI condition by itself, with little additional benefit being observed for reactive control, at least in the latencies.

As for the reason for this increased influence of contingency learning, it may be due to the fact that keypress responses to arrow directions, albeit not arbitrary, are not highly practiced responses (and in fact, to preview our discussion on time on task later in this section, those responses showed practice effects which vocal responses in Spinelli & Lupker’s (2020) original color-word Stroop experiment did not). The reason this fact is relevant is that contingency-learning effects are larger (and, therefore, potentially more influential) in experiments involving responses that are not well practiced (e.g., keypress responses to colors) than in experiments involving more practiced responses (e.g., vocal responses to colors: Forrin & MacLeod, 2017; Spinelli et al., 2020). In any case, if either (or both) of these proposals are correct, error rates would be the most appropriate dependent variable to examine in future research aimed at inducing reactive control with the present paradigm. With this consideration in mind, one suggestion would be to modify the paradigm in order for the research focus to be only on accuracy by, for example, using a fixed (Jacoby et al., 2003) or adaptive (Draheim et al., 2021) deadline for the response and examining the proportion of correct responses within that deadline.

Future research should also attempt to determine the reason why reactive-control contrasts similar to the one we used in the present experiment do tend to produce an effect in the latencies for color-word and picture-word Stroop tasks (e.g., Bugg & Hutchison, 2013; Bugg et al., 2011; Spinelli & Lupker, 2020). The presence of those effects would seem to imply that, in those experiments, even when the task goal is correctly maintained, responding to incongruent stimuli that are typically congruent still takes longer than responding to matched incongruent stimuli that are typically incongruent.

Two additional points are worth noting. The first is that, as with the list-wide PC effect for the diagnostic items in Experiment 1, the reliability of the reactive-control effect in Experiment 2 (i.e., the crucial contrast in this experiment) was poor, especially in the error rates (i.e., the dependent variable that produced the expected effect at the group level).

The second point concerns effects of time on task. For the type of design used in the present experiment, Spinelli and Lupker (2020) reported different time courses for reactive-control and contingency-learning effects in a color-word Stroop task, with the former growing over the course of the experiment and the latter remaining stable, consistent with previous research (Crump & Milliken, 2009; Jacoby et al., 2003; Schmidt et al., 2010). We ran the same analyses for the present Experiment 2 but could not replicate those results. In particular, in the RTs, the reactive-control effect was actually significantly smaller in the second block (in which MC stimuli were 10 ms *faster* than the matched MI stimuli) compared to the first block of the experiment (in which MC stimuli were 13 ms slower than the matched MI stimuli), whereas the contingency-learning effect was the same size in the two blocks (there was no change in the error rates across blocks for either effect). 

Also, different from Spinelli and Lupker’s (2020) experiment, RTs were overall 56 ms faster in the second block compared to the first block for the stimuli involved in the reactive-control contrast, a practice effect (also accompanied by no change in the error rates) that may partially explain the reduction of the effect in that contrast. In general, however, the point is that these results represent our third failure since Spinelli and Lupker (2020) to obtain evidence in the item-specific PC paradigm that reactive-control effects unconfounded from non-conflict effects grow over the course of the experiment (the other two failures are reported in Spinelli et al., 2022b). That particular result reported by Spinelli and Lupker (2020), therefore, does not appear to be robust. Future research should address the question of whether reactive-control effects do grow during the experiment using experiments involving more trials than the relevant experiments have typically used thus far.

In any case, overall, Experiment 2 demonstrated that it is possible to examine reactive control independently from non-conflict processes in a spatial Stroop task, although the reactive-control effect obtained does not seem to be as strong as the effect associated with proactive control obtained in Experiment 1.

## General discussion

### Summary and response to potential challenges

Adjusting control either proactively or reactively based on the situation are fundamental abilities in human cognition (Braver, 2012). Despite the research interest in proactive and reactive forms of conflict-induced control in recent years, examining these processes has proven to be somewhat challenging (Braem et al., 2019). Most common solutions for examining conflict-induced control independently from processes that, although unrelated to conflict, may produce similar patterns of results as conflict-based processes do, involve the use of relatively large sets of stimuli. Large sets of stimuli, however, are often inconvenient, because they typically require an equivalent number of responses that are difficult for researchers to collect in many situations (e.g., in neuroimaging and online experiments) or difficult for participants to learn when target-response mappings are arbitrary (e.g., manual responses to colors in the color-word Stroop task).

In the present research, we demonstrated the usefulness of a spatial Stroop task for solving many of these problems. We did so by implementing a variation of this task with six targets, six distractors, six responses, and Proportion-Congruent (PC) paradigms designed to examine proactive and reactive processes independently from non-conflict processes.

In Experiment 1, we focused on the list-wide PC effect, an effect associated with proactive control, and found that, for the diagnostic items (a fixed set of stimuli), the congruency effect in both RTs and error rates was much larger when those diagnostic items appeared in a list (the Mostly-Congruent list) in which they were intermixed with a separate set of mostly-congruent stimuli, the inducer items, compared to when the diagnostic items appeared in a list (the Mostly-Incongruent list) in which they were intermixed with mostly-incongruent inducer items. According to most researchers (e.g., Blais & Bunge, 2010; Bugg et al., 2008; Hutchison, 2011), this result can only be interpreted as evidence that selective attention to target information was regulated in a preparatory fashion in the MI list due to the high frequency of the conflict produced by the incongruent stimuli in the list, a proactive control process that would allow individuals to successfully select target information. In contrast, the low frequency of conflict in the MC list would induce little preparation for conflict, which would be dealt with reactively once it occurs. In sum, the list-wide PC effect produced by diagnostic items would indicate that the frequency of conflict in a list induces item-nonspecific, proactive control to be engaged.

According to some researchers (e.g., Algom & Chajut, 2019; Schmidt, 2013a, 2019), however, this evidence would still be insufficient to entirely rule out non-conflict processes as alternative explanations. The reason is that, although the fact that diagnostic items are identical in MC and MI lists when using inducer/diagnostic designs does prevent non-conflict processes such as contingency learning and repetition priming from explaining differences in performance for those stimuli in the two lists, those performance differences can, in theory, be produced by other non-conflict processes that may be at work in those designs.

One of those processes is a process whereby attention to distractors is increased in situations in which the distractors and targets used in the experiment are highly correlated, situations that occur when distractors and targets are not combined in a random fashion. In such situations, detecting the distractor (e.g., the word RED in the presented stimulus) makes it possible to form expectations about the target (e.g., that the most likely colors for RED are red and blue; Dishon-Berkovits & Algom, 2000; Sabri et al., 2001). It is often the case that this correlation, typically operationalized as C, a chi-square based contingency coefficient (Melara & Algom, 2003), is higher in MC lists than in MI lists, including in inducer/diagnostic designs (e.g., Bugg, 2014). This fact alone could explain why the former lists, that is, MC lists in which higher C values may attract attention to distractors, thus increasing the interference those distractors produce, tend to produce larger congruency effects than MI lists, even for diagnostic items (Algom & Chajut, 2019; Schmidt, 2019). Recently, however, Spinelli and Lupker (2023b) found no evidence in support of this idea in a series of experiments (see also Hasshim & Parris, 2021). In any case, this concern does not apply to our Experiment 1 because the design was set up so that arrows and locations would be correlated to the same degree in the two lists, as demonstrated by their equivalent C values, .88 (see also Spinelli & Lupker, 2021, 2023a).

Another non-conflict process that could explain differences for diagnostic items appearing in MC versus MI lists is a process whereby, in speeded tasks, participants form temporal expectancies for the emission of a response based on previous experience in the task and they then use those expectancies to guide their subsequent responses (Schmidt, 2013a). Specifically, in a list of trials in which there are many easy-to-process stimuli (e.g., congruent stimuli) such as an MC list, participants will form a fast temporal expectancy, which they can use to speed up responding to the easy-to-process stimuli if those stimuli have been processed enough for a likely correct response to be made at that point in time. The result would be an increased congruency effect in that situation. In contrast, in a list of trials in which there are many hard-to-process stimuli (e.g., incongruent stimuli) such as an MI list, participants will form a slow temporal expectancy, which they will use to respond faster than normal only to the hard-to-process stimuli for which a likely correct response can be made at around that point in time. The result would be a reduced congruency effect in that situation. That is, overall, this temporal-learning process could produce a list-wide PC effect by itself (for a demonstration of this possibility, see Schmidt, 2013a).

Controlling for temporal learning in the list-wide PC paradigm is a challenge because MC and MI lists differ intrinsically in overall ease of correct responding, and there is currently no agreed-upon analytical or experimental procedure to create such a control (Cohen-Shikora et al., 2019; Schmidt, 2017, 2022; Spinelli & Lupker, 2022). In general, however, Schmidt’s (2013a) temporal-learning account seems to have difficulty explaining how the typical observation in the list-wide PC paradigm, that latencies for the hard-to-process stimuli in that situation (i.e., the incongruent stimuli) tend to be faster in the “slow” list (i.e., the MI list, the list that creates the slower temporal expectancy) than in the “fast” list (i.e., the MC list), can be reconciled with the typical observation in other paradigms such as simple picture naming, that latencies for the hard-to-process stimuli in those situations (e.g., pictures that are hard to name) tend to be *slower* in “slow” lists (e.g., a list mostly composed of hard-to-name pictures) than in “fast” lists (e.g., a list mostly composed of easy-to-name pictures; see, e.g., Lupker et al., 1997, 2003; Spinelli et al., 2019). Until this contrast in the data patterns can be explained (for an initial discussion, see Schmidt, 2021), it remains unclear whether temporal learning actually does play a confounding role in the list-wide PC paradigm, at least for incongruent stimuli. (The slowdown for congruent stimuli in MI lists, on the other hand, might involve a temporal expectancy process slowing down latencies in those lists: Spinelli & Lupker, 2023a). Overall, therefore, the list-wide PC effect obtained for diagnostic items in the present Experiment 1 would seem more likely to index the impact of proactive control being engaged in the MI list rather than the impact of temporal expectancies.

There may, of course, be other non-conflict processes involved in our Experiment 1 that researchers in the area have not considered thus far and that may contribute to explaining the crucial (and rather large) list-wide PC effect that Experiment 1 produced. For example, there may be higher-order contingencies that participants could extract from the stimuli they were dealing with based on an understanding that the arrows and locations used were combined in pairs, and that one logical combination of the pair (e.g., the congruent one) was more likely than the other logical combination in the pair (e.g., the incongruent one) in a given list (e.g., the MC list). Participants might have thus learned that, in the MC list, the correct response would usually correspond with the location in which that arrow appeared (e.g., if the arrow appeared in the east location, the correct response would likely be the east one), whereas in the MI list, the correct response would usually correspond to the location that, in the relevant pair, was opposite to that in which the arrow appeared (e.g., if the arrow appeared in the east location, the correct response would likely be the west one, as west and east locations formed the relevant pair in that case). The result would be a list-wide PC effect not only for the inducer items (perhaps so strong for those items as to lead even to the reversal of the congruency effect in the MI list, as we have observed), but also for the diagnostic items. However, non-conflict processes of this type have yet to be formalized, which they would need to be before they can be seriously thought of as providing viable accounts.^5^ Until then, because our Experiment 1 abides by most of Braem et al.’s (2019) recommendations (with one exception, which we justify in the next section), a control explanation based on the idea that proactive control is engaged in the MI list appears to be the best interpretation for the crucial contrast examined in that experiment.

Similarly, reactive control would appear the best interpretation for the effect obtained in the contrast examined in Experiment 2 between incongruent stimuli belonging to a stimulus set associated with infrequent conflict, the MC set, and incongruent stimuli belonging to a stimulus set associated with frequent conflict, the MI set. The reason is that the stimuli involved in that contrast were matched on contingency learning and individual stimulus frequency, the main non-conflict factors that have been argued to play a role in the item-specific PC paradigm (e.g., Hazeltine & Mordkoff, 2014; Schmidt & Besner, 2008; note that correlation-based and time-based processes have not been argued to play a role in this paradigm, unlike in the list-wide PC paradigm). Thus, at present, the only viable explanation of the fact that the MI incongruent stimuli elicited fewer errors than the matched MC incongruent stimuli would be that selective attention to target information was reactively increased upon presentation of the former stimuli compared to the latter. The fact, however, that this effect only emerged in the error rates seems to suggest that the main impact of that process in our task was to cause fewer goal-neglect episodes for MI stimuli than for MC stimuli, as discussed above.

As was also discussed above, the present design does not allow a determination of the trigger of reactive control. Reactive control could be triggered by recognition of either the individual target, the individual distractor, and/or, more generally, the side of the stimulus (left vs. right). Those distinctions were not of primary importance for the present research in which we aimed to measure reactive control regardless of its trigger. Researchers who are interested in those distinctions may, however, use alternative, albeit more complex, designs such as that used by Spinelli et al. (2022b), which make examinations of some of the relevant distinctions possible.

Finally, we noted, in passing, that while Experiment 1 produced a very large effect in its crucial contrast (the proactive, item-nonspecific contrast), the same cannot be said for Experiment 2 (the reactive, item-specific contrast). Barring explanations based on non-conflict processes such as those discussed thus far and those discussed in the next section, this overall pattern of results may suggest that in the spatial Stroop task that we used, compared to other relevant tasks in the literature, control adjustments may be based less on item-specific information, such as the congruency proportion associated with individual targets and/or distractors, and more on general information, such as the congruency proportion associated with a list as a whole. The reason might have to do with the fact that the stimuli used in this task are perceptually similar to one another (i.e., arrows only changing in orientation and location of presentation) and differences among them might be difficult to encode and/or retrieve. Future research should attempt to examine this idea more closely, perhaps by comparing item-nonspecific and item-specific effects across tasks involving highly similar versus dissimilar stimuli (see, e.g., Bugg & Dey, 2018; Cochrane & Pratt, 2022b).

### Direct and indirect impacts of non-conflict processes

Although the present experiments produced evidence for proactive and reactive control processes independently from non-conflict processes, evidence was also produced (in different contrasts) suggesting that non-conflict processes do play a role. In Experiment 1, a reversed congruency effect in the latencies was observed in the MI list for inducer items. As noted, the most likely explanation for that pattern is that contingency learning and/or repetition-priming, processes that were not controlled for in the inducer items, facilitated responses to incongruent stimuli in the MI list to the point of making those stimuli faster than congruent stimuli.

In Experiment 2, an impact of contingency learning and/or repetition priming was not simply inferred but was observed in a contrast between stimuli that were otherwise matched. That is, the contrast was between incongruent stimuli which required their typical response and were highly frequent in the experiment and other incongruent stimuli which required what was, for them, an atypical response and were relatively infrequent in the experiment, with the former stimuli showing shorter latencies and higher accuracy. Overall, the implication of these findings is that manipulating the proportion of congruent and incongruent stimuli in an experiment engages not only control processes but also non-conflict ones (Spinelli & Lupker, 2023a). Therefore, when the research interest lies on the former processes, controlling the latter processes with designs such as those used in the present experiments becomes especially important (Braem et al., 2019).

There is also another, less direct way in which non-conflict processes can have an impact on experiments such as the present ones. According to Bugg (2014) and Bugg et al. (2011), the availability of contingency learning in MI conditions in list-wide and item-specific PC paradigms will determine what type of process will be the dominant one (i.e., the one used most frequently in order to minimize interference in those conditions): either contingency learning itself, when this process is available in MI conditions (i.e., when each distractor in those conditions can be associated with a specific incongruent target/response), or proactive/reactive control (in list-wide/item-specific PC paradigms, respectively), when contingency learning is not available in MI conditions (i.e., when no distractor in those conditions can be easily associated with a single specific incongruent target/response). Essentially, the idea is that a non-conflict process such as contingency learning will not be merely additive with conflict-induced ones (Schmidt & Besner, 2008) but its availability in MI conditions may actually determine whether conflict-induced processes are used at all in those conditions. This type of idea has also resulted in the recommendation for research on conflict-induced control to focus on paradigms that do not make contingency learning a viable process for minimizing interference in MI conditions (Braem et al., 2019).^6^ Because that recommendation is possibly the only recommendation of Braem et al. (2019) that we have *not* followed (i.e., contingencies between distractors and incongruent targets/responses could be learned in the MI conditions in our experiments), readers might wonder why.

In response, we would like to note that, first, Braem et al.’s (2019) recommendation is based on a premise that has turned out to be false, i.e., that in situations in which contingency learning *is* a viable process to minimize interference in MI conditions, conflict-induced control will never be used. There are now a few demonstrations that this premise is false in both the list-wide paradigm (Schmidt, 2017; Spinelli & Lupker, 2023a) and the item-specific paradigm (Spinelli & Lupker, 2020; Spinelli et al., 2020, 2022b), in addition to those produced by the present experiments.

Second, applying that recommendation actually results in failing to do a complete job of controlling for all non-conflict processes that have been presumed to contribute to PC effects. For example, in the list-wide PC paradigm, the target-distractor correlation value, C, discussed above, is inevitably higher in MC lists than in MI lists when applying Braem et al.’s recommendation, opening up the possibility that a process of adjusting attention to that correlation, as opposed to a conflict-induced process, would be the one producing the list-wide PC effect in that situation (for similar arguments concerning the list-wide as well as the item-specific PC paradigm, see Schmidt, 2019).

Third and finally, as Schmidt (2019) noted, contrary to Braem et al.’s recommendation, most of the published experiments in the literature *did* make contingency learning a viable process to minimize interference in MI conditions. Therefore, to maintain contact with the bulk of the literature, it would seem more appropriate for researchers to continue the study of conflict-induced control in situations in which that form of control is one of the options available to participants for reducing interference in the MI conditions as opposed to focusing exclusively on situations in which conflict-induced control is the only such option, as Braem et al.’s recommendation appears to imply. It is for these reasons that, in the present experiments, we opted not to follow that recommendation.

That said, Braem et al.’s (2019) recommendation may have some merits. In particular, while the present experiments make it clear that it is not necessary to follow that recommendation in order for some evidence of conflict-induced control to be found in this task, following it may result in that evidence becoming stronger because, in that situation, conflict-induced control would be the only option for participants to use in order to reduce interference in MI conditions. For example, as noted in the *Discussion* section of Experiment 2, the impact of reactive control might have been felt not only in the error rates but also in the latencies in that experiment had contingency learning not produced such a large effect for that dependent variable.

Further, the task presented here does allow modifications which would allow interested researchers to implement Braem et al.’s (2019) recommendation, at least for the list-wide PC manipulation (for the item-specific PC manipulation, the situation appears to be somewhat more complex).^7^ For example, rather than using two inducer subsets with a set size of two for a list-wide PC manipulation, researchers may want to use a single inducer subset with a set size of four (e.g., rather than presenting the north-east-pointing arrow only in the north-east and south-west location as we have done in Experiment 1, that arrow could also be presented in north-west and south-east locations as well; note that the diagnostic arrows would still be presented in two locations). Doing so would allow researchers to construct an MI list in which, following Braem et al.’s recommendation, the inducer stimuli would not make contingency learning a viable process to reduce interference in that list because each arrow in that subset would appear equally frequently in each of the four locations associated with that subset (the congruent location and the three incongruent ones).^8^

### Limitations

The present experiments were not intended to address more general concerns in the literature about conflict-induced control. One such concern is that because PC manipulations typically involve only congruent and incongruent stimuli with no neutral baseline (e.g., a colored letter string in the color-word Stroop task), it is impossible to determine whether it is mainly facilitation or interference (produced by congruent and incongruent stimuli, respectively, compared to neutral stimuli), or both, that drives the observed PC effects, and whether those manipulations affect facilitation and interference in a similar fashion (Algom et al., 2022). Another concern is that by assuming a generic “conflict” that incongruent but not congruent stimuli would produce, PC manipulations typically neglect important differences between conflict components (including the fact that congruent stimuli would not be completely conflict-free; Parris et al., 2021). These concerns naturally apply to the present experiments as well. Further, it is unlikely that spatial Stroop tasks could ever be used to fully address those types of concerns because dissociating facilitation and conflict components requires several control conditions (e.g., neutral conditions) that are hard to implement in those tasks (e.g., there would appear to be only one usable neutral distractor, a central location).

Research using other tasks, particularly color-word Stroop tasks, however, does provide some support for the idea that interference plays a strong role in conflict-induced control (Spinelli & Lupker, 2021; Tzelgov et al., 1992), with response conflict (i.e., conflict arising from competing responses) potentially being the key component, as the tasks in which this type of conflict is smaller (e.g., manual, compared to vocal, color-word Stroop tasks; Augustinova et al., 2019) are also the tasks producing smaller PC effects, if those effects emerge at all (Bejjani et al., 2020; Bejjani & Egner, 2021; Blais & Bunge, 2010). Considering the similarities between spatial and color-word Stroop tasks (Lu & Proctor, 1995; Viviani et al., 2023), it is reasonable to assume that interference, created, in particular, by response conflict, is the driving force of the conflict-induced control effects reported in the present experiments. However, these ideas are still speculative and will need to be examined more extensively in future research.

Indeed, it may be the case that, although (confound-controlled) PC effects involve conflict-induced control, conflict (of any kind) may not actually play the major role in those processes. Instead, those processes may reflect an adaptation to the response specified by the distractor (when that response can be processed early enough) rather than the conflict that the distractor creates, with that response being favored in situations in which that response is often correct (i.e., MC conditions) and disfavored in situations in which that response is often incorrect (i.e., MI conditions; Weissman et al., 2015, in review). Although the distinction between conflict-based and response-based control is not a large one, adjudicating between the two accounts is another issue that future research will need to address.

One potential way of addressing this issue for researchers who require a task that is easy to implement such as the present one, but flexible enough to include, for example, several neutral conditions, would be to resort to color-word or picture-word Stroop tasks involving typed responses (Crump et al., 2017; Logan & Zbrodoff, 1998). A starting point would be to replicate our results in a typed-response color-word Stroop task based on the designs of the present Experiments 1 and 2.

These considerations, along with the fact that our experiments produced patterns of results that do not completely overlap with those typically produced by other Stroop tasks (i.e., the reversed congruency effect for inducer items in Experiment 1, the reactive-control effect emerging in the error rates but not the RTs in Experiment 2), should make it clear that although our spatial Stroop task belongs to the family of Stroop tasks, it should not be considered a perfect substitute for any other task in that family. Clearly, each task has its own characteristics, strengths, and weaknesses, that researchers must be aware of when using them.

In any case, the present research, overall, would seem to make a strong case that a spatial Stroop task like the one we used may be an effective tool in research on conflict-induced control. Using this task, it is possible to: (1) have a stimulus set large enough to examine proactive and reactive control processes independently from non-conflict processes; (2) collect data with ease even outside the laboratory; and (3) avoid presenting participants with a challenging experimental setup. Additionally, the non-verbal nature of the stimuli can allow researchers to address empirical questions for which it is preferable that the stimuli not be verbal (e.g., research involving participants with varying linguistic abilities).

Note, however, that we do not mean to suggest that the present task could not be improved or that it would be useful in all situations. For example, it may not always be comfortable for participants to use the six response keys that we used in the present experiments. An alternative option might be to require participants to respond with a single finger which would be held in a central position at the beginning of the trial and moved to the required key afterwards. This type of response might also be made, especially in laboratory experiments, with movements made with a mouse, a joystick, or on a touchscreen.

Further, in the present form, our task might not be appropriate for individual-differences research. In line with the “reliability paradox” (Hedge et al., 2018), the effect associated with the highest reliability among the crucial contrasts examined in the present experiments, i.e., the reactive-control effect in the latencies in Experiment 2 (*r*_SB_ = .39, a value that is still not a high one in absolute terms), was the only null effect at the group level. As noted, part of the reason for these poor reliabilities is that the crucial contrasts involve difference scores. The implication is that the effects produced by those contrasts are not very stable at the individual level, meaning that they may have limited utility for research on individual differences. One possibility for increasing the utility of this task for that type of research might be to modify it in order to make it produce a single score, such as the average time taken to complete a list of trials (Draheim et al., 2021), although it is unclear how this modification would allow a contrast of MC and MI conditions.

Despite these (very common) limitations, the spatial Stroop task that we presented has considerable potential, especially in experimental research, for examining proactive and reactive control as recommended by Braem et al. (2019) and it is hoped that future research will consider it seriously for that purpose.

## Footnotes

1 Since Stroop (1935) introduced the standard, color-word version of the task, several other versions have been developed that are commonly called “Stroop” or “Stroop-like” tasks despite involving, in some cases, neither words or colors. Here, we rely on Kornblum’s (1992) model for a taxonomy of “ensembles”, or types of interference tasks (i.e., tasks involving stimuli which involve an easily processed irrelevant component – the distractor – that needs to be ignored and a less easily processed relevant component – the target – that requires, most typically, an identification response). Following Kornblum’s model, we will refer to our task as well as any other interference task that, regardless of the materials involved, would be classified as a type-8 ensemble, that is, one in which representations for distractors, targets, and responses overlap, as a “Stroop task.” For example, a picture-word interference task involving vocal responses (Lupker, 1979) meets this definition because, for example, the representation for the word “dog” overlaps with that for the image of a dog as well as with that for the response “dog.” However, as discussed below, other interference tasks, such as “Stroop-like” and “Simon” tasks, do not meet this definition despite the fact that the materials involved might be similar.

2 Upon inspection of the 61 PET/fMRI articles including at least one Stroop experiment listed in the meta-analysis articles by Laird et al. (2005) and Huang et al. (2020), we only found eight of them that reported having recorded overt vocal responses during the scanning session, many of which are PET studies from the late 1990s/early 2000s (Carter et al., 1995, 2000; George et al., 1994, 1997; Kronhaus et al., 2006; MacDonald et al., 2000; Ravnkilde et al 2002; Taylor et al., 1997). Although collecting vocal responses in neuroimaging research is not impossible (see also Braver et al., 2021), doing so certainly poses a challenge.

3 We used a lower cutoff of 300 ms to be consistent with prior work of ours with several Stroop tasks, including the particular spatial Stroop task used in the present experiments (Spinelli et al., 2022a, b). However, that cutoff may be felt to be a bit slow considering that RTs under 300 ms can be observed in choice reaction time tasks (Welford, 1980). Further, the specific cutoff one chooses might make a difference, especially for reliability (Parsons, 2022). Therefore, the ANOVA, t-tests, and reliability analyses for the crucial contrasts in both experiments were repeated using a 200-ms lower cutoff. The patterns of results were virtually identical, including those for the reliability analyses.

4 We focused on the list-wide PC effect produced by diagnostic items because, as noted, it is the crucial effect in our manipulation. However, list-wide PC manipulations have also been used for the purpose of examining the reliability of the congruency effect in MC and MI lists (as opposed to the list-wide PC effect which is the difference between congruency effects in MC vs. MI lists; Borgmann et al., 2007). We conducted such an analysis for the congruency effect produced by diagnostic items in the MC list and, separately, the MI list. We focused on diagnostic items only for this analysis because they are confound-free and because we deemed that inducer items involved too few observations in the infrequent cells to conduct such an analysis, i.e., only eight incongruent stimuli in the MC list and only eight congruent ones in the MI list. The Spearman-Brown corrected split-half reliabilities for the congruency effect were, for the latencies, *r*_SB_ = .46, 95% CI [-.10, .73] in the MC list and *r*_SB_ = .62, 95% CI [.38, .78] in the MI list; for the error rates, they were *r*_SB_ = .70, 95% CI [.54, .82] in the MC list and *r*_SB_ = .21, 95% CI [-.22, .55] in the MI list. Thus, while the reliability of the congruency effect was better for the MI list than the MC list in the latencies, it was better for the MC list than the MI list in the error rates. Further, note that, although those reliability values would be deemed acceptable, none was particularly high.

5 Proper consideration of non-conflict processes of this type would also likely require a different design than that used in the present Experiment 1 in order to dissociate those processes from conflict-induced ones. In the present design, what might offer some insight is an examination of the errors committed on congruent stimuli, particularly for diagnostic items in the MI list. If participants were biased to produce opposite-side responses in that list (e.g., for an arrow presented in the “east” location, they were biased to respond “west”), those responses, contrasted with non-opposite-side error responses (e.g., “north-west”, “south-west”, “north-east”, or “south-east” responses when the correct response was “east”), should have been disproportionately represented among the errors committed on congruent stimuli in that list, even for diagnostic items. Note, however, that those responses only represented only 43% of the errors in that condition in Experiment 1 (with the remaining 57% representing non-opposite-side responses). Of course, one might argue that 43% is still higher than the proportion that would have been expected due to chance (i.e., with five possible incorrect responses, chance would be 20%). However, the proportion of opposite-side responses was, at 30%, higher than chance even for the congruent diagnostic items in the MC list, a list in which there should have been no bias for opposite-side responses. In any case, these data need to be taken with caution because they were derived from only 37 and 33 data points in the MI and MC list, respectively (as is typically the case, errors to congruent stimuli were few in number).

6 To be more precise, Braem et al. (2019) explicitly made this recommendation for the list-wide PC manipulation only. In discussing experiments which attempted to control for non-conflict processes in the item-specific PC manipulation, Braem et al. focused only on experiments in which the item-specific manipulation was based on the identity of the targets as opposed to the identity of the distractors (e.g., color-word Stroop experiments in which the colors, not the words, were manipulated in order to be either MC or MI). However, since those experiments originated from Bugg et al. (2011), who used that type of design precisely to prevent contingency learning from being used to reduce interference in the MI condition and who first recommended that that type of design should be used to examine reactive control in the item-specific PC manipulation, it would seem safe to assume that Braem et al.’s position is consistent with that recommendation.

7 The difficulty comes from the fact that Braem et al.’s (2019) recommendation involves a distinction between inducer and diagnostic stimuli that the present Experiment 2 does not make. However, that distinction is made in Spinelli et al.’s (2022b) Experiment 2, a spatial Stroop task such as the present one. Readers who are interested in applying Braem et al.’s recommendation to the item-specific PC paradigm using the present task are thus referred to that article. Even so, implementing that recommendation may not be trivial because it would seem to involve a somewhat major modification of the design of that experiment, a modification for which we are currently unable to provide useful pointers.

8 However, do note that constructing such a list while maintaining the total frequency of each arrow and location as equivalent for all items, as we have done for the present experiments and recommend (for a discussion, see Spinelli & Lupker, 2023a), would make the list-wide congruency proportion of the resulting list 37.50%, which is a bit higher than is typical for MI lists. The list-wide PC manipulation might thus end up being weak unless that MI list is contrasted with an MC list with a very high list-wide congruency proportion (e.g., 75%, if all inducer stimuli are made congruent and none were incongruent). Alternatively, or in addition, the list-wide congruency proportion of the MI list could be reduced by decreasing the number of congruent stimuli within the inducer set (as done by, e.g., Bugg and Gonthier, 2020) or eliminating them altogether (i.e., making all inducer stimuli incongruent and none congruent), with the latter option bringing the list-wide congruency proportion down to 16.67%, a more typical proportion for an MI list.

## Declarations

### Open practices statement

The raw data, the aggregated data in SPSS format, the JASP and R files, and study materials are available at https://osf.io/6v2p9/. The study was not preregistered.
